# Supplementary material for: Molecular design strategy of fluorogenic probes based on quantum chemical prediction of intramolecular spirocyclization
Source: Commun Chem. 2020 Jun 26;3:82. doi: 10.1038/s42004-020-0326-x (PMC9814528; doi:10.1038/s42004-020-0326-x)
Supplement: Supplementary file 1 — Supplementary Information [file 42004_2020_326_MOESM1_ESM.pdf]

Supplementary Information

Molecular Design Strategy of Fluorogenic Probes Based on Quantum Chemical Prediction of Intramolecular Spirocyclization

Ryo Tachibana, Mako Kamiya, Satoshi Suzuki, Keiji Morokuma, Aika Nanjo, Yasuteru Urano.

Supplementary Methods

Reagents

General chemicals were of the best grade available, supplied by Aldrich Chemical Co., Ltd., Tokyo Chemical Industries, and Wako Pure Chemical, and were used without further purification. Special chemicals: dimethyl sulfoxide (from DOJINDO) used in spectroscopic analysis was of fluorometric grade. Other solvents were used after appropriate distillation or purification.

Instruments

NMR spectra were recorded on a JEOL JNM-LA300 instrument at 300 MHz for ^{1}H NMR and 75 MHz for ^{13}C NMR or on a JEOL JNM-LA400 instrument at 400 MHz for ^{1}H NMR and 100 MHz for ^{13}C NMR. All chemical shifts (δ) are reported in ppm relative to internal standard tetramethylsilane (δ = 0.0 ppm), or relative to the signals of residual solvent $CDCl_3$ (7.26 ppm for ^{1}H, 77.16 ppm for ^{13}C), CD_3OD (3.31 ppm for ^{1}H, 49.00 ppm for ^{13}C) or DMSO-d_6 (2.50 ppm for ^{1}H, 39.52 ppm for ^{13}C), and coupling constants are given in Hz. Mass spectra were measured with a JEOL AccuTOF 4GLC-plus mass spectrometer for ESI^+ and ESI^-. HPLC analyses were performed on an Inertsil ODS-3 (4.5×250 mm) column (GL Sciences Inc.) using an HPLC system composed of a pump (PU-2080, JASCO) and a detector (MD-2015, JASCO). Preparative HPLC was performed with an Inertsil ODS-3 (10.0 mm ×250 mm) reverse-phase column (GL Science Inc.), using eluent A (H_2O containing 0.1% TFA (v/v)) and eluent B (CH_3CN with 20% H_2O), on a Jasco PU-1587S2 system. Silica gel column chromatography was performed with Wakogel C-200 (Wako, Japan), Chromatorex-NH (FujiSilysia Chemical Ltd., Kasugai, Japan), silica Gel 60 (Kanto Chemical Co. Inc., Tokyo, Japan), or silica gel 60N (Kanto Chemical Co. Inc., Tokyo, Japan). Absorbance spectral measurements were obtained on a Shimadzu UV-1800.

Synthesis of HMR derivatives

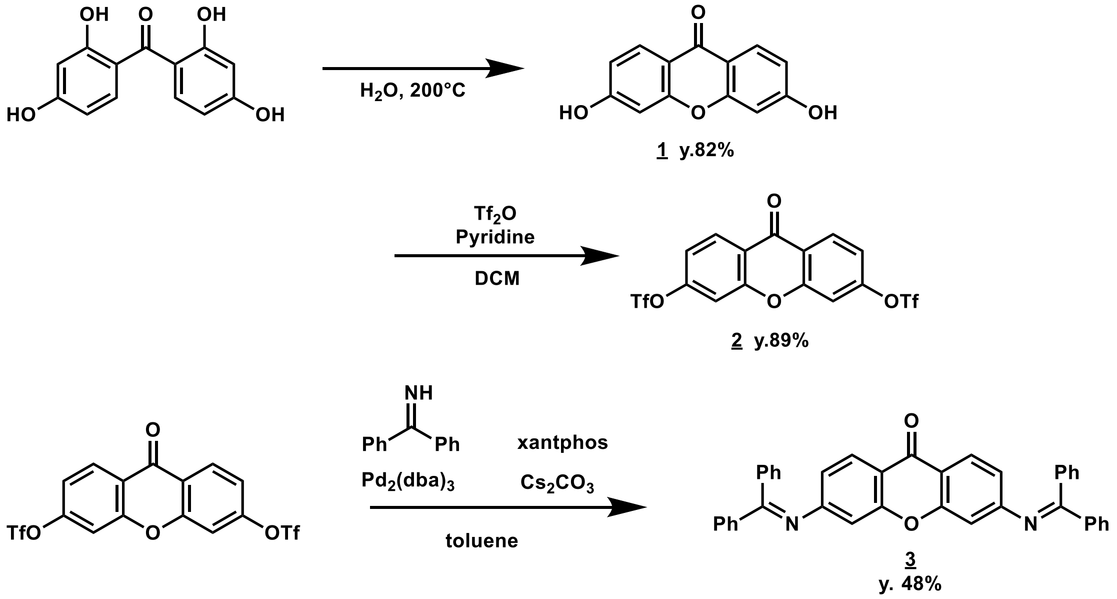

1

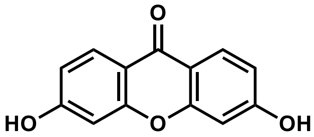

2,2',4,4'-Tetrahydroxybenzophenone (4500 mg, 18.3 mmol) in H_2O (12 ml) was heated at 200 °C for 3 hr with microwave. After cooling, the product was filterd, washed with cold hexane and dried to give compound 1 as a pale brown solid (3410 mg, 82%). ^{1}H NMR (300 MHz, DMSO-*d6*): δ 6.78-6.88 (m, 4H), 7.99 (d, 2H, J = 9.0 Hz), 10.81 (br, 2H). ^{13}C NMR (75 MHz, DMSO-*d6*): δ 102.0, 113.6, 114.0, 127.7, 157.4, 163.3, 173.9.

2

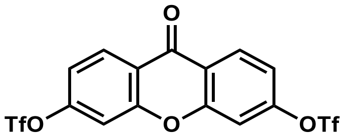

Compound 1 (1300 mg, 5.7 mmol) was added to 40 ml CH_2Cl_2 and pyridine (4.6 mL, 57 mmol) was added. The mixture was stirred at 0 °C for 10 min, then Tf_2O (2.8 mL, 17.1 mmol) was added dropwise. The reaction mixture was warmed to room temperature and stirred for 4 hr. The reaction was quenched with H_2O and extracted with CH_2Cl_2. The organic solution was dried over Na_2SO_4, filtered, and evaporated. The residue was purified by flash column chromatography (silica gel, CH_2Cl_2) to give compound 2 as a colorless solid (2492 mg, 89%).^{1}H NMR (300 MHz, $CDCl_3$): δ 7.36 (dd, 2H, J = 8.7 Hz, 2.1 Hz), 7.50 (d, 2H, J = 2.1 Hz), 8.45 (d, 2H, J = 8.7 Hz). ^{13}C NMR (100 MHz, $CDCl_3$): δ 111.5, 118.2, 118.7 (q, J = 320 Hz), 121.4, 129.7, 153.4, 156.6, 174.6

3

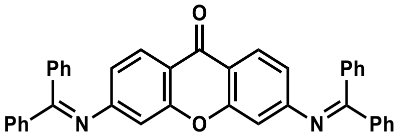

Compound 2 (1000 g, 2.0 mmol), benzophenone imine (3.4 mL, 20.3 mmol), $Pd_2(dba)_3 \cdot CHCl_3$ (207 mg, 0.2 mmol), xantphos (289 mg, 0.5 mmol) and Cs_2CO_3 (6614 g, 20.3 mmol) were dissolved in toluene (10 mL) under an argon atmosphere. The resulting mixture was stirred at 80 °C overnight, and then cooled to room temperature. The reaction mixture was diluted with H_2O and extracted with AcOEt. The organic layer was collected and evaporated to dryness. The residue was purified by column chromatography (silica gel, hexane/AcOEt=7/3) to give compound 3 as a yellow solid (538 mg, 48%). ^{1}H NMR (300 MHz, $CDCl_3$): δ 6.66 (dd, 2H, J = 8.4 Hz, 1.8 Hz), 6.74 (d, 2H, J = 2.2 Hz), 7.03 (m, 4H), 7.29–7.57 (m, 12H), 7.78–7.82 (m, 4H), 8.05 (d, 2H, J = 8.8 Hz). ^{13}C NMR (100 MHz, $CDCl_3$): δ 108.3, 117.5, 117.5, 127.2, 128.2, 128.3, 129.3, 129.6, 131.3, 135.4, 138.8, 156.8, 157.4, 169.2, 176.0.

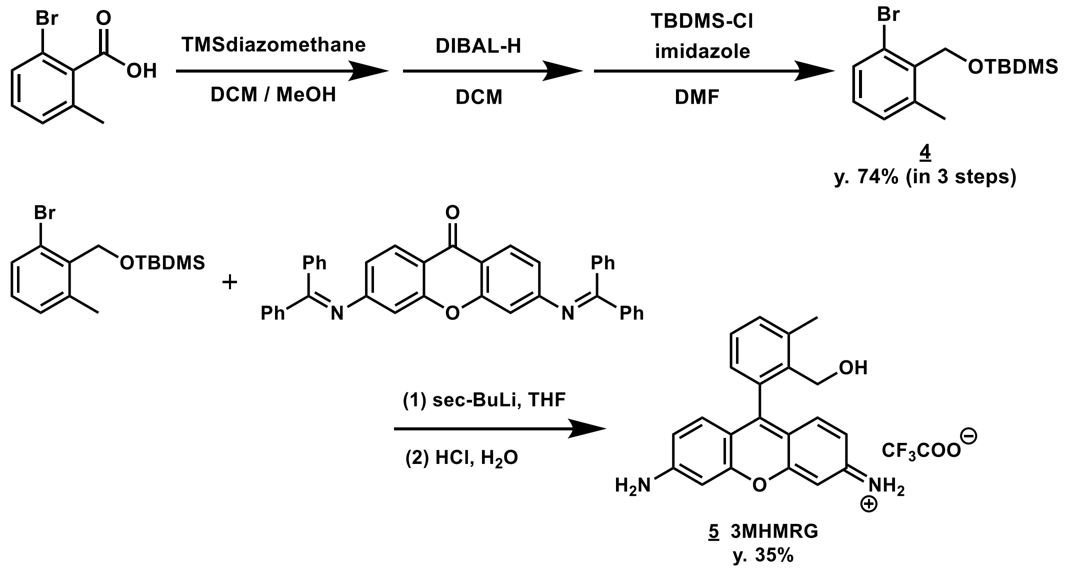

4

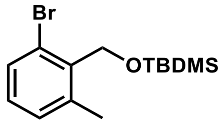

2-Bromo-6-methylbenzoic acid (2000 mg, 9.3 mmol) was dissolved in CH_2Cl_2 (20 mL) and methanol (6 mL). Trimethylsilyldiazomethane (18.6 mL, 11.2 mmol) was added and the solution was stirred at r.t. for 10 minutes. The reaction was quenched with AcOH and the mixture was extracted with CH_2Cl_2 from saturated $NaHCO_3$ aq.. The organic solution was dried over Na_2SO_4, filtered, and evaporated. The residue was dissolved in anhydrous CH_2Cl_2 (20 mL), and the resulting solution was stirred at 0˚C under an Ar atmosphere. Diisobutylaluminium hydride (18.6 mL, 18.6 mmol) was added and stirring was continued at r.t. under an Ar atmosphere for 2 hours. The reaction was quenched with methanol and the mixture was extracted with CH_2Cl_2 from saturated potassium sodium tartrate aq.. The organic solution was dried over Na_2SO_4, filtered, and evaporated. The residue was dissolved in anhydrous DMF (20 mL). tert-Butyldimethylchlorosilane (1402 mg, 9.3 mmol) and imidazole (1266 mg, 18.6 mmol) were added and the solution was stirred at r.t. under an Ar atmosphere for 4 hours. The mixture was extracted with CH_2Cl_2. The organic solution was dried over Na_2SO_4, filtered, and evaporated. The residue was purified by flash column chromatography (silica gel, *n*-hexane/AcOEt = 9/1 to 7/3) to give compound 4 as a colorless liquid (2169 mg, 74%). ^{1}H NMR (400 MHz, $CDCl_3$): δ 0.12 (s, 6H), 0.92 (s, 9H), 2.46 (s, 3H), 4.88 (s, 2H), 7.01 (dd, 1H, *J* = 8.1 Hz, 8.1 Hz), 7.10 (d, 1H, *J* = 7.8 Hz), 7.38 (d, 1H, *J* = 7.8 Hz). ^{13}C NMR (75 MHz, $CDCl_3$): δ -5.2, 18.4, 19.9, 25.9, 63.1, 125.2, 128.9, 129.7, 130.5, 137.6, 140.4.

5 3MHMRG

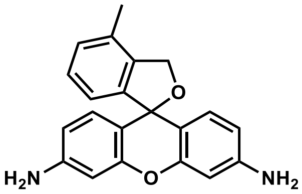

To a flame-dried flask flushed with Ar, compound 4 (851 mg, 2.7 mmol) and anhydrous THF (7 mL) were added. The mixture was cooled to -90˚C and then 1 M *sec*-BuLi (2.6 mL, 2.7 mmol) was added to it. Compound 3 (150 mg, 0.27 mmol) in anhydrous THF (5 mL) was further added, and the mixture was stirred at -90˚C for 10 minutes, then at r.t. for 4 hours. The reaction was quenched with 2 N HCl aq. and the mixture was extracted with CH_2Cl_2 from saturated $NaHCO_3$ aq.. The organic solution was dried over Na_2SO_4, filtered, and evaporated. The residue was purified by preparative HPLC under the following conditions: A/B = 80/20 (0 min) to 0/100 (30 min) linear gradient (solvent A: H_2O, 0.1% TFA; solvent B: acetonitrile/H_2O = 80/20, 0.1% TFA). Compound 5 was obtained as an orange solid (31 mg, 35%). ^{1}H NMR (400 MHz, CD_3OD+NaOD): δ 2.36 (s, 3H), 5.16 (s, 2H), 6.39 (dd, 2H, J = 8.4 Hz, 2.4 Hz) 6.47 (d, 2H, J = 2.4 Hz), 6.59 (s, 1H), 6.60 (d, 2H, J = 8.4 Hz), 7.14-7.19 (m, 2H). ^{13}C NMR (100 MHz, CD_3OD+NaOD): δ 18.5, 71.6, 86.5, 102.2, 112.5, 115.3, 122.1, 129.6, 130.8, 132.2, 139.2, 145.8, 150.3, 153.0, 170.2. HRMS (ESI$^+$): Calcd for $[M+H]^+$, 331.14465, Found, 331.14731 (+2.66 mmu)

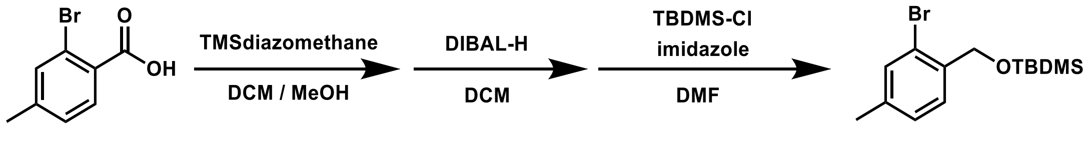

6
y. 81% (in 3 steps)

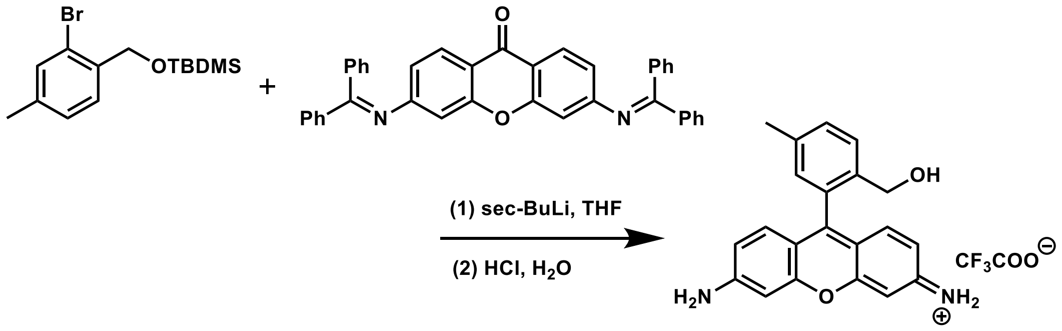

7 3MHMRG
y. 45%

6

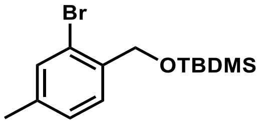

2-Bromo-4-methylbenzoic acid (2000 mg, 9.3 mmol) was dissolved in CH_2Cl_2 (45 mL) and methanol (15 mL). Trimethylsilyldiazomethane (17 mL, 10.2 mmol) was added and the solution was stirred at r.t. for 30 minutes. The reaction was quenched with AcOH and the mixture was extracted with CH_2Cl_2 from saturated $NaHCO_3$ aq.. The organic solution was dried over Na_2SO_4, filtered, and evaporated. The residue was dissolved in anhydrous CH_2Cl_2 (50 mL), and the solution was stirred at 0˚C under an Ar atmosphere. Diisobutylaluminium hydride (18.6 mL, 18.6 mmol) was added and stirring was continued at r.t. under an Ar atmosphere for 6 hours. The reaction was quenched with methanol and the mixture was extracted with CH_2Cl_2 from saturated potassium sodium tartrate aq.. The organic solution was dried over Na_2SO_4, filtered, and evaporated. The residue was dissolved in anhydrous DMF (40 mL). tert-Butyldimethylchlorosilane (2110 mg, 14.0 mmol) and imidazole (1900 mg, 27.9 mmol) were added and the solution was stirred at r.t. under an Ar atmosphere for 20 hours. The mixture was extracted with CH_2Cl_2. The organic solution was dried over Na_2SO_4, filtered, and evaporated. The residue was purified by flash column chromatography (silica gel, *n*-hexane) to give compound 6 as a colorless liquid (2374 mg, 81%). ^{1}H NMR (400 MHz, $CDCl_3$): δ 0.12 (s, 6H), 0.96 (s, 9H), 2.31 (s, 3H), 4.71 (s, 2H), 7.12 (d, 1H, *J* = 8.0 Hz), 7.32 (s, 1H), 7.41 (d, 1H, *J* = 8.0 Hz). ^{13}C NMR (100 MHz, $CDCl_3$): δ -5.2, 18.5, 20.8, 26.1, 64.6, 120.9, 127.5, 128.2, 132.6, 137.3, 138.3.

7 5MHMRG

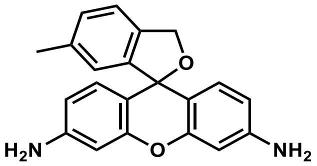

To a flame-dried flask flushed with Ar, compound 6 (568 mg, 1.8 mmol) and anhydrous THF (15 mL) were added. The mixture was cooled to -78˚C and then 1 M *sec*-BuLi (1.4 mL, 1.4 mmol) was added to it. Compound 3 (100 mg, 0.18 mmol) in anhydrous THF (4 mL) was further added, and the mixture was stirred at -78˚C for 10 minutes, then at r.t. for 1 hour. The reaction was quenched with 2 N HCl aq. and the mixture was extracted with CH_2Cl_2 from saturated $NaHCO_3$ aq.. The organic solution was dried over Na_2SO_4, filtered, and evaporated. The residue was purified by preparative HPLC under the following conditions: A/B = 80/20 (0 min) to 0/100 (45 min) linear gradient (solvent A: H_2O, 0.1% TFA; solvent B: acetonitrile/H_2O = 80/20, 0.1% TFA). Compound 7 was obtained as an orange solid (27 mg, 45%).^{1}H NMR (400 MHz, CD_3OD+NaOD): δ 2.22 (s, 3H), 5.11 (s, 2H), 6.37 (dd, 2H, *J* = 8.8 Hz, 2.4 Hz) 6.45 (d, 2H, *J* = 2.4 Hz), 6.56 (s, 1H), 6.57 (d, 2H, *J* = 8.8 Hz), 7.15 (d, 1H, *J* = 8.0 Hz), 7.24 (d, 1H, *J* = 8.0 Hz). ^{13}C NMR (100 MHz, CD_3OD+NaOD): δ 20.0, 70.8, 84.5, 100.8, 111.2, 113.9, 120.2, 123.8, 128.7, 129.5, 136.1, 138.0, 145.2, 149.0, 151.7. HRMS (ESI$^+$): Calcd for $[M+H]^+$, 331.14465, Found, 331.14144(-3.21 mmu)

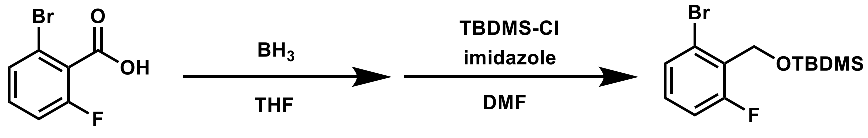

8
y. 63% (in 2 steps)

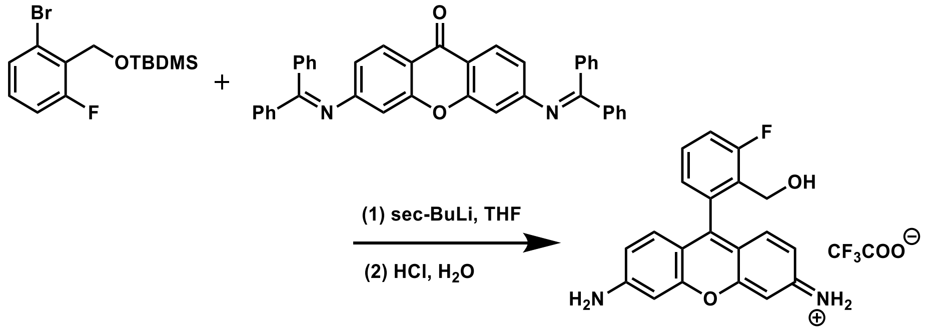

9 3FHMRG
y. 48%

8

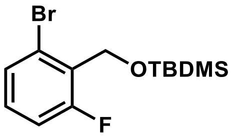

2-Bromo-6-fluorobenzoic acid (1000 mg, 4.57 mmol) was dissolved in CH_2Cl_2 (10 mL) and methanol (3 mL). Trimethylsilyldiazomethane (8.4 mL, 5.02 mmol) was added and the solution was stirred at r.t. for 30 minutes. The reaction was quenched with AcOH and the mixture was extracted with CH_2Cl_2 from saturated $NaHCO_3$ aq.. The organic solution was dried over Na_2SO_4, filtered, and evaporated. The residue was dissolved in anhydrous CH_2Cl_2 (20 mL), and the solution was stirred at 0˚C under an Ar atmosphere. Diisobutylaluminium hydride (9.1 mL, 9.14 mmol) was added and stirring was continued at r.t. under an Ar atmosphere for 3 hours. The reaction was quenched with methanol and the mixture was extracted with CH_2Cl_2 from saturated potassium sodium tartrate aq.. The organic solution was dried over Na_2SO_4, filtered, and evaporated. The residue was dissolved in anhydrous DMF (10 mL). tert-Butyldimethylchlorosilane (1033 mg, 6.86 mmol) and imidazole (933 mg, 13.7 mmol) were added, and the solution was stirred at r.t. under an Ar atmosphere for 4 hours. The mixture was extracted with CH_2Cl_2. The organic solution was dried over Na_2SO_4, filtered, and evaporated. The residue was purified by flash column chromatography (silica gel, *n*-hexane/AcOEt = 10/0 to 8/2) to give compound 8 as a colorless liquid (920 mg, 63%).^{1}H NMR (300 MHz, $CDCl_3$): δ 0.12 (s, 6H), 0.91 (s, 9H), 4.83 (s, 2H), 7.01 (dd, 1H, *J* = 8.1 Hz, 8.1 Hz), 7.13 (d, 1H, *J* = 8.1 Hz), 7.36 (d, 1H, *J* = 8.1 Hz). ^{13}C NMR (75 MHz, $CDCl_3$): δ -5.3, 18.4, 25.9, 58.8, 114.8 (d, *J* = 23.0 Hz), 125.9 (d, *J* = 5.0 Hz), 127.9 (d, *J* = 17.4 Hz), 128.6 (d, *J* = 3.8 Hz), 130.0 (d, *J* = 9.9 Hz), 161.5 (d, *J* = 250.7 Hz).

9 3FHMRG

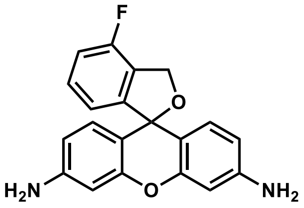

To a flame-dried flask flushed with Ar, compound 8 (230 mg, 0.72 mmol) and anhydrous THF (5 mL) were added. The mixture was cooled to -90˚C and then 1 M *sec*-BuLi (0.7 mL, 0.72 mmol) was added to it. Compound 3 (80 mg, 0.14 mmol) in anhydrous THF (5 mL) was further added, and the mixture was stirred at -90˚C for 10 minutes, then at r.t. for 18 hours. The reaction was quenched with 2 N HCl aq. and the mixture was extracted with CH_2Cl_2 from saturated $NaHCO_3$ aq.. The organic solution was dried over Na_2SO_4, filtered, and evaporated. The residue was purified by preparative HPLC under the following conditions: A/B = 80/20 (0 min) to 0/100 (30 min) linear gradient (solvent A: H_2O, 0.1% TFA; solvent B: acetonitrile/H_2O = 80/20, 0.1% TFA). Compound 9 was obtained as an orange solid (23 mg, 48%). ^{1}H NMR (400 MHz, CD_3OD+NaOD): δ 5.23 (s, 2H), 6.40 (dd, 2H, *J* = 8.4 Hz, 2.0 Hz), 6.46 (d, 2H, *J* = 2.0 Hz), 6.61 (d, 2H, *J* = 8.4 Hz), 6.61 (d, 1H, *J* = 8.4 Hz), 7.08 (dd, 1H, *J* = 8.0 Hz, 8.4 Hz), 7.30 (m, 1H). HRMS (ESI$^+$): Calcd for $[M+H]^+$, 335.11958, Found, 335.11932 (-0.26 mmu).

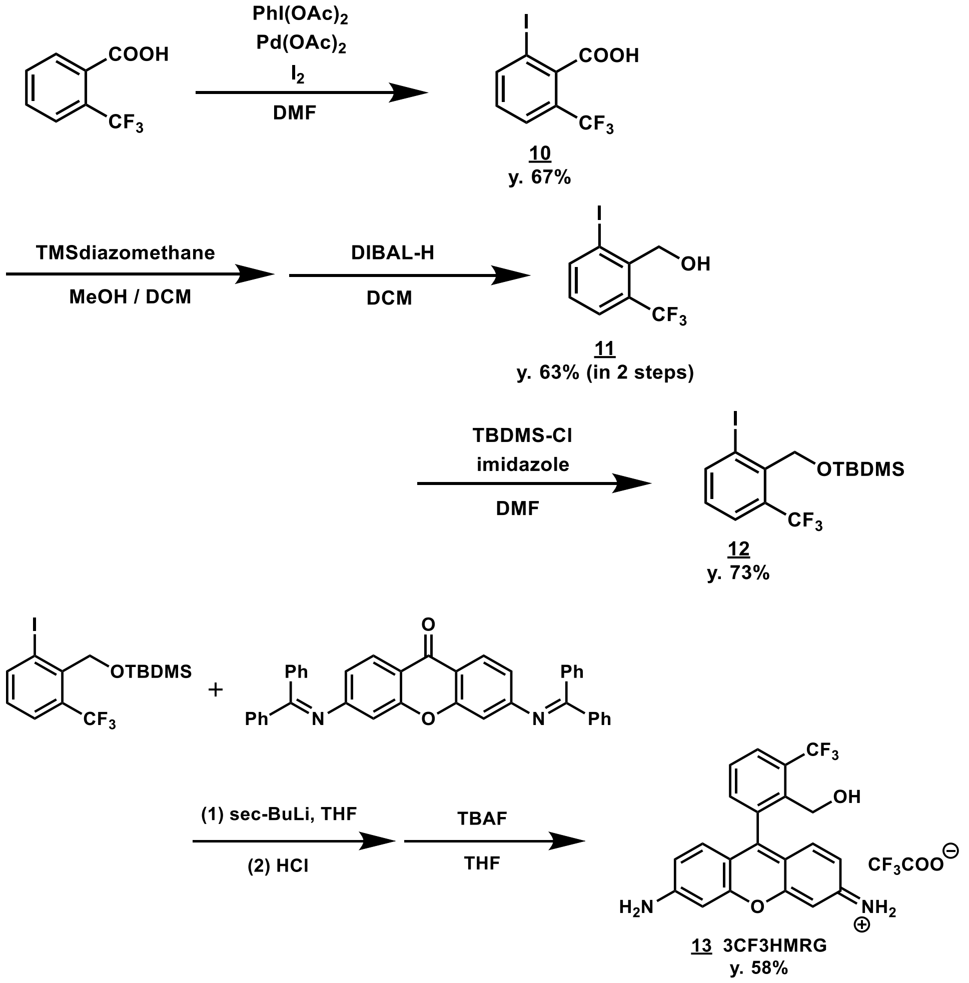

10

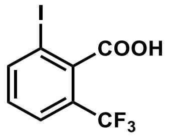

o-Trifluoromethylbenzoic acid (2000 mg, 10.5 mmol), iodobenzene diacetate (3382 mg, 10.5 mmol), palladium(II) acetate (119 mg, 0.53 mmol) and iodine (2665 mg, 10.5 mmol) were dissolved in anhydrous DMF (40 mL) and the solution was stirred at 100˚C under an Ar atmosphere for 24 hours. The reaction was quenched with saturated Na_2SO_3 aq. and the mixture was acidified with 2 N HCl aq., then extracted with CH_2Cl_2. The organic solution was dried over Na_2SO_4, filtered, and evaporated. The residue was purified by flash column chromatography (silica gel, *n*-hexane/AcOEt containing 2% AcOH = 7/3 to 5/5) to give compound 10 as a colorless solid (2206 mg, 67%). ^{1}H NMR (300 MHz, DMSO-d_6): δ 7.39 (dd, 1H, J = 7.3 Hz, 7.3 Hz), 7.83 (d, 1H, J = 7.3 Hz), 8.22 (d, 1H, J = 7.3 Hz), 13.8-14.3 (br, 1H). ^{13}C NMR (75 MHz, DMSO-d_6): δ 59.7, 94.5, 125.8, 125.8, 130.9, 143.2, 168.1, 170.3.

11

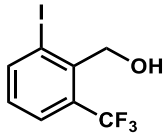

Compound 10 (1500 mg, 4.75 mmol) was dissolved in CH_2Cl_2 (20 mL) and methanol (6 mL). Trimethylsilyldiazomethane (9.5 mL, 5.70 mmol) was added and the solution was stirred at r.t. for 4 hours. The reaction was quenched with AcOH and the mixture was extracted with CH_2Cl_2 from saturated $NaHCO_3$ aq.. The organic solution was dried over Na_2SO_4, filtered, and evaporated. The residue was dissolved in anhydrous CH_2Cl_2 (20 mL), and the solution was stirred at 0˚C under an Ar atmosphere. Diisobutylaluminium hydride (9.5 mL, 9.50 mmol) was added and stirring was continued at r.t. under an Ar atmosphere for 4 hours. The reaction was quenched with methanol and the mixture was extracted with CH_2Cl_2 from saturated potassium sodium tartrate aq.. The organic solution was dried over Na_2SO_4, filtered, and evaporated. The residue was purified by flash column chromatography (silica gel, *n*-hexane/AcOEt = 93/7 to 72/28) to give compound 11 as a colorless liquid (900 mg, 63%). ^{1}H NMR (300 MHz, $CDCl_3$): δ 2.16 (t, 1H, *J* = 7.3 Hz), 4.90 (d, 2H, *J* = 7.3 Hz), 7.12 (dd, 1H, *J* = 8.1 Hz, 8.1 Hz), 7.68 (d, 1H, *J* = 8.1 Hz), 8.10 (d, 1H, *J* = 8.1 Hz). ^{13}C NMR (75 MHz, DMSO-d_6): δ 60.4, 65.2, 103.7, 126.2, 126.3, 129.6, 143.8, 171.1.

12

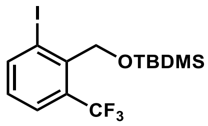

Compound 11 (800 mg, 2.65 mmol), tert-butyldimethylchlorosilane (598 mg, 3.97 mmol) and imidazole (361 mg, 5.30 mmol) were dissolved in anhydrous DMF (10 mL), and the solution was stirred at r.t. under an Ar atmosphere for 9 hours. The mixture was extracted with CH_2Cl_2. The organic solution was dried over Na_2SO_4, filtered, and evaporated. The residue was purified by flash column chromatography (silica gel, *n*-hexane/AcOEt = 100/0 to 87/13) to give compound 12 as a colorless liquid (800 mg, 73%). ^{1}H NMR (300 MHz, $CDCl_3$): δ 0.17 (s, 6H), 0.94 (s, 9H), 4.86 (s, 2H), 7.07 (dd, 1H, *J* = 8.1 Hz, 8.1 Hz), 7.65 (d, 1H, *J* = 8.1 Hz), 8.08 (d, 1H, *J* = 8.1 Hz). ^{13}C NMR (100 MHz, $CDCl_3$): δ -3.5, 25.7, 53.4, 93.7, 125.9, 130.8, 142.8, 167.4.

13 3CF3HMRG

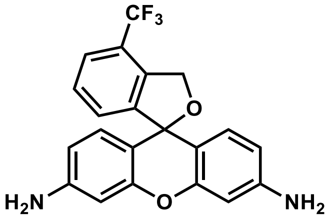

To a flame-dried flask flushed with Ar, compound 12 (300 mg, 0.72 mmol) and anhydrous THF (5 mL) were added. The mixture was cooled to -90˚C and then 1 M *sec*-BuLi (0.7 mL, 0.72 mmol) was added to it. Compound 3 (80 mg, 0.14 mmol) in anhydrous THF (5 mL) was further added, and the mixture was stirred at -90˚C for 10 minutes, then at r.t. for 4 hours. The reaction was quenched with 2 N HCl aq. and the mixture was extracted with CH_2Cl_2 from saturated $NaHCO_3$ aq.. The organic solution was dried over Na_2SO_4, filtered, and evaporated. The residue was dissolved in anhydrous THF (10 mL). Tetrabutylammonium fluoride (0.25 mL) was added and the solution was stirred at r.t. for 2 hours. The residue was purified by preparative HPLC under the following conditions: A/B = 80/20 (0 min) to 0/100 (30 min) linear gradient (solvent A: H_2O, 0.1% TFA; solvent B: acetonitrile/H_2O = 80/20, 0.1% TFA). Compound 13 was obtained as an orange solid (32 mg, 58%). ^1H NMR (400 MHz, CD_3OD): δ 5.31 (s, 2H), 6.39 (dd, 2H, *J* = 8.8 Hz, 2.2 Hz), 6.46 (d, 2H, *J* = 2.2 Hz), 6.55 (d, 2H, *J* = 8.8 Hz), 7.06 (d, 1H, *J* = 8.1 Hz), 7.46 (dd, 1H, *J* = 8.1 Hz, 8.1 Hz), 7.66 (d, 1H, *J* = 8.1 Hz). HRMS (ESI^+): Calcd for $[M+H]^+$, 385.11639, Found, 385.11343 (+2.96 mmu)

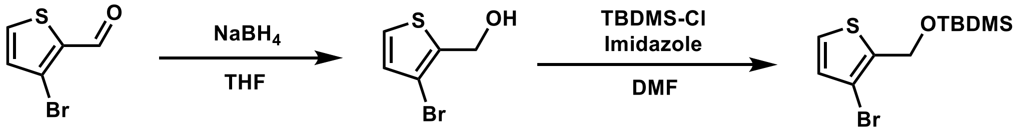

14 y.quant

15 y.91%

14

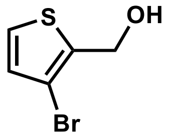

3-Bromothiophene-2-carboxaldehyde (1910 mg, 10.0 mmol) was dissolved in anhydrous THF (40 mL) and the solution was stirred at 0˚C. Sodium tetrahydroborate (757 mg, 20.0 mmol) was added and stirring was continued at r.t. for 3 hours. The reaction was quenched with 2 N HCl aq. and the mixture was extracted with CH_2Cl_2. The organic solution was dried over Na_2SO_4, filtered, and evaporated. The residue was purified by flash column chromatography (silica gel, *n*-hexane/AcOEt = 9/1 to 7/3) to give compound 14 as a colorless liquid (2015 mg, quant.). ^{1}H NMR (300 MHz, $CDCl_3$): δ 2.25 (t, 1H, *J* = 5.1 Hz), 4.79 (d, 2H, *J* = 5.1 Hz), 6.96 (d, 1H, *J* = 5.9 Hz), 7.27 (d, 1H, *J* = 5.9 Hz).

15

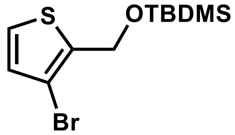

Compound 14 (2000 mg, 10.36 mmol), tert-butyldimethylchlorosilane (2342 mg, 15.54 mmol) and imidazole (2116 mg, 31.08 mmol) were dissolved in anhydrous DMF (20 mL), and the solution was stirred at r.t. under an Ar atmosphere for 3 hours. The mixture was extracted with *n*-hexane from brine. The organic solution was dried over Na_2SO_4, filtered, and evaporated. The residue was purified by flash column chromatography (silica gel, *n*-hexane) to give compound 15 as a colorless liquid (2884 mg, 91%). ^{1}H NMR (400 MHz, $CDCl_3$): δ 0.11 (s, 6H), 0.93 (s, 9H), 4.80 (s, 2H), 6.90 (d, 1H, *J* = 4.8 Hz), 7.20 (d, 1H, *J* = 4.8 Hz). ^{13}C NMR (100 MHz, $CDCl_3$): δ -5.2, 18.4, 25.9, 90.7, 106.1, 124.6, 129.8, 140.4.

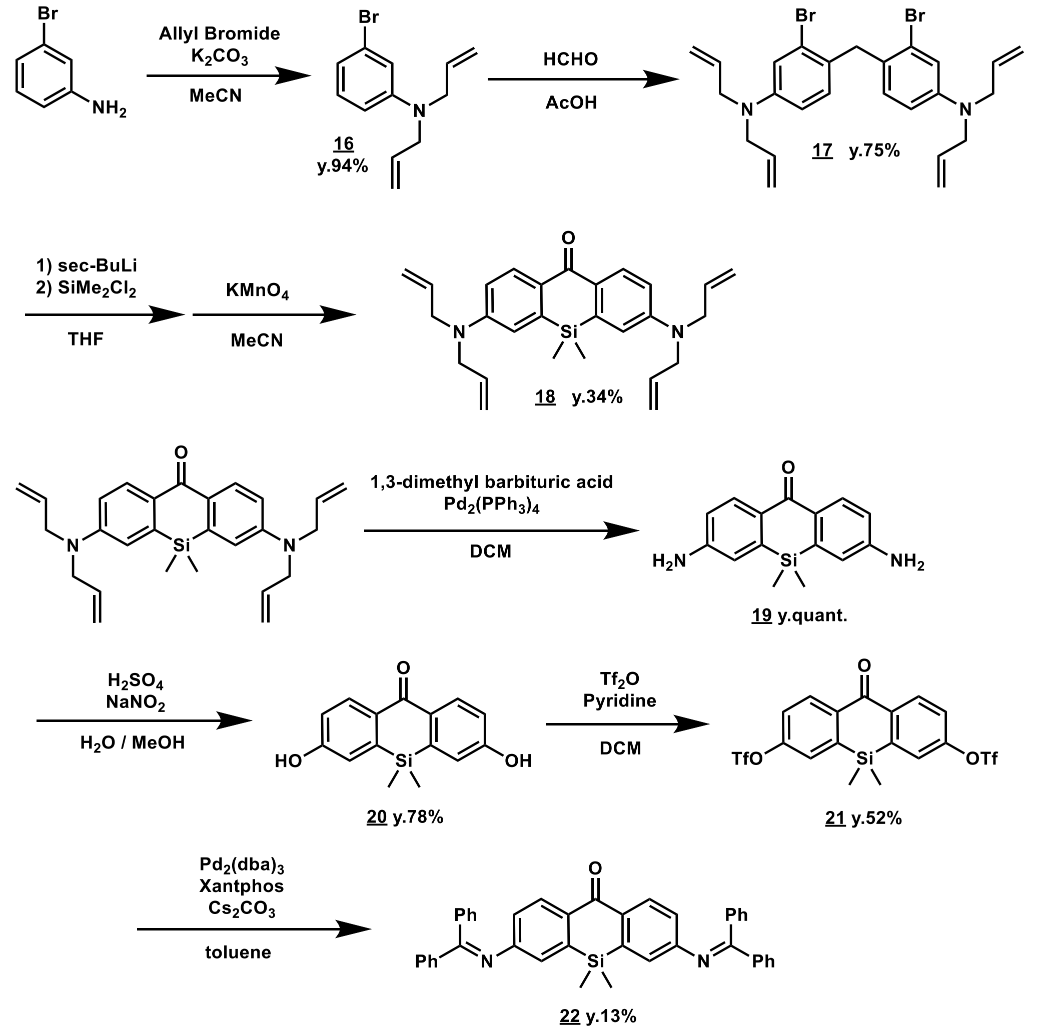

Br
NH2
Allyl Bromide
K2CO3
MeCN
Br
N
16
y.94%
HCHO
AcOH
Br
Br
N
N
17 y.75%
1) sec-BuLi
2) SiMe2Cl2
THF
KMnO4
MeCN
O
N
Si
N
18 y.34%
O
N
Si
N
1,3-dimethyl barbituric acid
Pd2(PPh3)4
DCM
O
H2N
Si
NH2
19 y.quant.
H2SO4
NaNO2
H2O / MeOH
O
HO
Si
OH
20 y.78%
Tf2O
Pyridine
DCM
O
TfO
Si
OTf
21 y.52%
Pd2(dba)3
Xantphos
Cs2CO3
toluene
O
Ph
Ph
N
Si
N
Ph
Ph
22 y.13%

16

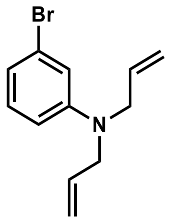

3-bromoaniline (12.7 mL, 116.3 mmol), K_2CO_3 (32.1 g, 232.5 mmol) and allyl bromide (29.5 mL, 348.8 mmol) were added in MeCN (100 mL), and the mixture was refluxed at 100 °C for 21 h. After cooling, the reaction mixture was filtered, extracted with CH_2Cl_2 and evaporated. The residue was purified by column chromatography (silica gel, hexane/AcOEt=9/1) to give compound 16 as colorless liquid (27.5 g, 94%). ^{1}H NMR (300 MHz, $CDCl_3$): δ 3.87-3.90 (m, 4H), 5.11-5.15 (m, 2H), 5.17-5.18 (m, 2H), 5.75-5.88 (m, 2H) 6.58 (dd, 1H, *J* = 2.2, 8.1 Hz), 6.77-6.81 (m. 2H) 7.01 (t, 1H, *J* = 8.1Hz); ^{13}C NMR (75 MHz, $CDCl_3$): δ 52.7, 110.8, 115.0, 116.3, 119.0, 123.3, 130.2, 133.2, 150.0.

17

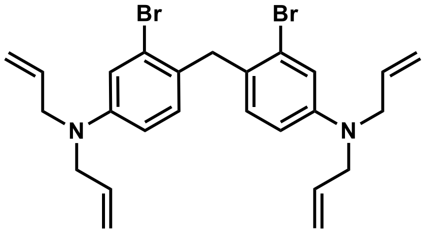

To a solution of compound 16 (25.0 g, 99.1 mmol) in AcOH (100 mL) was added 37% formaldehyde (8.9 mL, 119 mmol), and the mixture was stirred at 80 °C for 2 hr. After cooling to room temperature, the reaction mixture was neutralized with saturated $NaCO_3$ aq., and extracted with CH_2Cl_2. The organic layer was washed with brine, dried over Na_2SO_4 and evaporated to dryness. The residue was purified by column chromatography (silica gel, n-hexane/AcOEt = 97/3) to give compound 17 as colorless liquid (19.1 g, 75%). ^{1}H NMR (300 MHz, $CDCl_3$): δ 3.85-3.87 (m, 8H), 3.96 (s, 2H), 5.13-5.19 (m, 8H), 5.76-5.88 (m, 4H), 6.54(dd, 2H, *J* = 2.9, 8.8 Hz), 6.81 (d, 2H, *J* = 8.1 Hz), 6.90 (d, 2H, *J* = 2.9 Hz); ^{13}C NMR (75 MHz, $CDCl_3$): δ 39.7, 52.7, 111.7, 116.0, 116.2, 125.5, 126.9, 130.8, 133.5, 148.1.

18

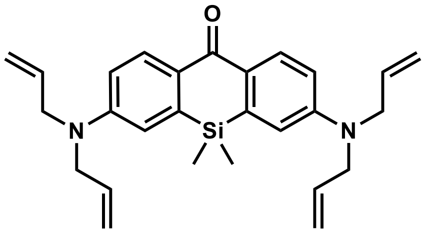

To a flame-dried flask flushed with argon, compound 17 (18.0 g, 34.9 mmol) and anhydrous THF (50 mL) were added. The solution was cooled to –78 °C, 1 M sec-BuLi (87.2 mmol) was added, and the mixture was stirred for 15 min. Then $SiMe_2Cl_2$ (8.3 mL, 69.7 mmol) was slowly added. The mixture was warmed to room temperature, then stirred for 2 hr. The reaction was quenched by addition of 2 N HCl aq., then the mixture was neutralized with $NaHCO_3$, and extracted with CH_2Cl_2. The organic layer was washed with brine, dried over Na2SO4 and evaporated to dryness. The residue was dissolved in MeCN (100 mL), and the solution was cooled to 0 °C. To this solution, $KMnO_4$ (16.5 g, 104.6 mmol) was added in small portions over a period of 2 h with stirring. The mixture was stirred for another 2.5 h at the same temperature, then quenched with MeOH, filtered through celite and evaporated to dryness. The residue was purified by column chromatography (silica gel, n-hexane/CH_2Cl_2 = 1/1 to 0/1) to give compound 18 as yellow solid (5.09 g, 34%). ^{1}H NMR (300 MHz, $CDCl_3$): δ 0.41 (s, 6H), 4.02 (d, 8H, J = 5.1 Hz) 5.17-5.23 (m, 8H), 5.82-5.94 (m, 4H), 6.80-6.83 (m, 4H), 8.34 (d, 2H, J = 8.1 Hz); ^{13}C NMR (75 MHz, $CDCl_3$): δ -1.1, 52.8, 113.5, 114.8, 116.7, 130.0, 131.7, 133.1, 140.5, 150.2, 185.1.

19

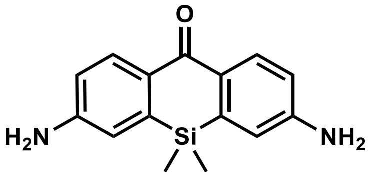

Compound 18 (5000 mg, 11.7 mmol), $Pd(PPh_3)_4$ (2704 mg, 2.34 mmol) and 1,3-dimethylbarbituric acid (9115 mg, 58.4 mmol) were added in CH_2Cl_2 (40 mL). The solution was stirred at 35 °C for 22 h, then evaporated to dryness. The residue was suspended in saturated Na_2CO_3 aq. and extracted with Cl_2Cl_2. The organic layer was washed with brine, dried over Na_2SO_4 and evaporated to dryness. The residue was purified by column chromatography (silica gel, n-hexane/AcOEt = 1/1 to 1/4) to give compound 19 as pale yellow solid (3400 mg, quant.). ^{1}H NMR (300 MHz, CD_3OD): δ 0.40 (s, 6H), 6.76 (dd, 2H, J = 2.6, 8.4 Hz), 6.88 (d, 2H, J = 2.2 Hz), 8.13 (d, 2H, J = 8.8 Hz); ^{13}C NMR (75 MHz, CD_3OD): δ -1.3, 116.6, 118.4, 131.0, 132.8, 142.6, 153.0, 187.5.

20

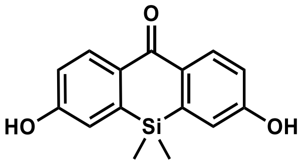

A solution of compound 19 (3400 mg, 12.67 mmol) in MeOH / 6 N H_2SO_4 (30 mL / 30 mL) was cooled to 0 °C. A solution of $NaNO_2$ (5244 mg, 76.0 mmol) in H_2O (10 mL) was slowly added, and the mixture was stirred at the same temperature for 15 min, then slowly added dropwise into boiling 1 N H_2SO_4 (180 mL). The resulting mixture was refluxed at 120 °C for another 20 min, then allowed to cool to room temperature, and extracted with CH_2Cl_2. The organic layer was washed with brine, dried over Na_2SO_4 and evaporated. The residue was purified by column chromatography (silica gel, n-hexane/AcOEt = 3/2 to 1/1) to give compound 6 as pale yellow solid (2670 mg, 78%). ^{1}H NMR (300 MHz, CD_3OD): δ 0.45 (s, 6H), 6.95 (dd, 2H, J = 2.2, 8.8 Hz), 7.07 (d, 2H, J = 2.2 Hz), 8.26 (d, 2H, J = 8.8 Hz); ^{13}C NMR (75 MHz, CD_3OD): δ -1.5, 118.4, 120.0, 133.3, 133.8, 143.1, 162.2, 187.6.

21

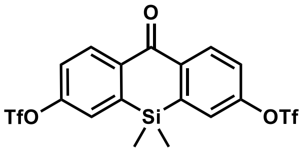

Compound 20 (1600 mg, 5.92 mmol) and pyridine (1.9 mL, 23.7 mmol) were dissolved in anhydrous CH_2Cl_2 (40 mL) and the mixture was stirred at 0˚C. Then, trifluoromethanesulfonic anhydride (3.9 mL, 23.7 mmol) was added, and stirring was continued for 4 hours. The reaction was quenched with H_2O and the mixture was extracted with CH_2Cl_2. The organic solution was dried over Na_2SO_4, filtered, and evaporated. The residue was purified by flash column chromatography (silica gel, CH_2Cl_2) to give compound 21 as a colorless solid (1660 mg, 52%). ^{1}H NMR (400 MHz, $CDCl_3$): δ 0.55 (s, 6H), 7.48 (dd, 2H, J = 2.8 Hz, 8.8 Hz), 7.57 (d, 2H, J = 2.8 Hz), 8.49 (d, 2H, J = 8.8 Hz). ^{13}C NMR (100 MHz, $CDCl_3$): δ -2.14, 118.8 (q, J = 320 Hz), 123.2, 125.6, 132.9, 140.0, 142.2, 152.2, 184.8.

22

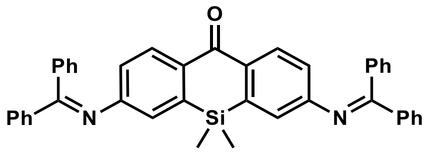

Compound 21 (1500 mg, 2.8 mmol), benzophenone imine (4060 mg, 22.4 mmol), $Pd_2(dba)_3$ (513 mg, 0.56 mmol), xantphos (324 mg, 0.56 mmol) and Cs_2CO_3 (9123 mg, 28.0 mmol) were dissolved in deaerated dioxane (50 mL), and the solution was stirred at 100˚C under Ar atmosphere for 22 hours. The mixture was extracted with CH_2Cl_2 and the organic solution was dried over Na_2SO_4, filtered, and evaporated. The residue was purified by flash column chromatography (silica gel, *n*-hexane/AcOEt = 10/0 to 7/3) to give compound 22 as a yellow solid (220 mg, 13%). ^{1}H NMR (400 MHz, CD_2Cl_2): δ 0.13 (s, 6H), 6.82 (d, 2H, *J* = 2.4 Hz), 6.95 (dd, 2H, *J* = 8.4, 2.4 Hz), 7.11-7.15 (m, 3H), 7.22-7.32 (m, 6H), 7.42-7.53 (m, 7H), 7.78 (d, 4H, *J* = 8.0 Hz), 8.20 (d, 2H, *J* = 8.4 Hz). ^{13}C NMR (100 MHz, CD_2Cl_2) δ -1.64, 123.1, 125.2, 128.4, 128.6, 129.2, 129.6, 129.8, 130.3, 130.6, 131.5, 136.3, 139.3, 140.2, 154.6, 169.2, 186.1. HRMS (ESI$^+$): calcd for $[M+H]^+$, 597.23621 ; found, 597.23370 (-2.51 mmu).

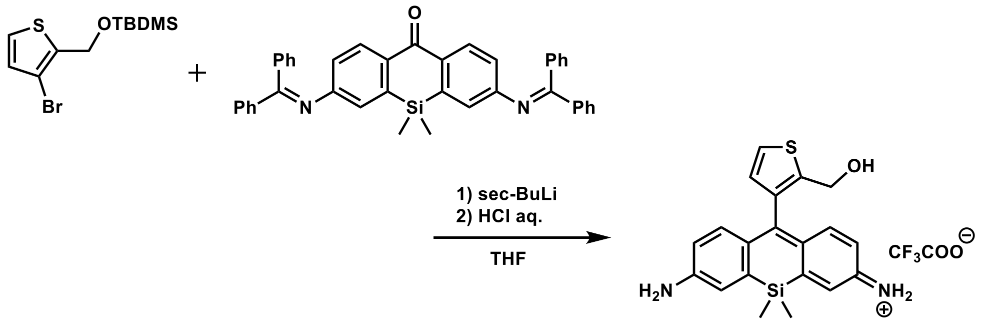

23 HM3ThPSiR600
y.92%

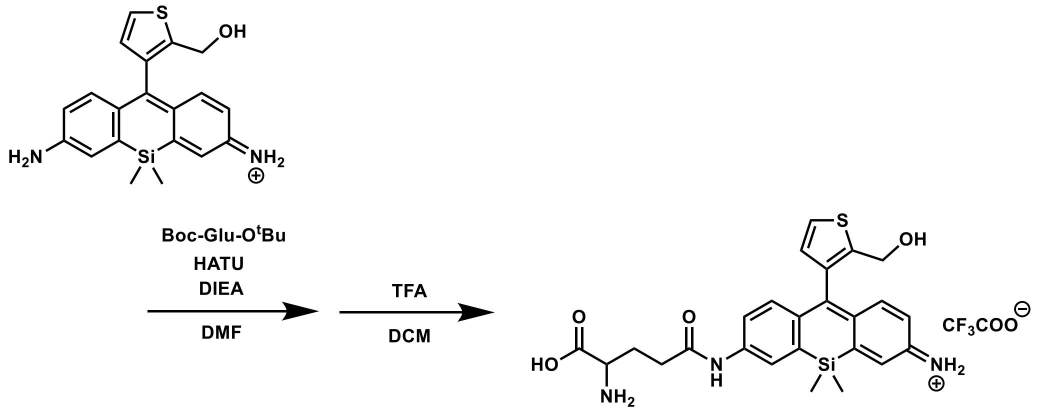

24 gGlu-HM3ThPSiR600
y.80%

23 HMRR (HM3ThPSiR600)

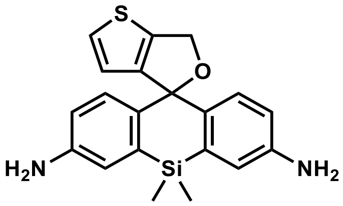

To a flame-dried flask flushed with Ar, compound 15 (412 mg, 1.34 mmol) and anhydrous THF (10 mL) were added. The mixture was cooled to -78˚C and then 1 M *sec*-BuLi (1.3 mL, 1.30 mmol) was added to it. Compound 22 (80 mg, 0.13 mmol) in anhydrous THF (4 mL) was further added, and the mixture was stirred at r.t. for 1 hour. The reaction was quenched with 2 N HCl aq. and the mixture was extracted with CH_2Cl_2 from saturated $NaHCO_3$ aq.. The organic solution was dried over Na_2SO_4, filtered, and evaporated. The residue was purified by preparative HPLC under the following conditions: A/B = 80/20 (0 min) to 0/100 (30 min) linear gradient (solvent A: H_2O, 0.1% TFA; solvent B: acetonitrile/H_2O = 80/20, 0.1% TFA). Compound 23 was obtained as an orange solid (50 mg, 92%). ^{1}H NMR (300 MHz, CD_3OD+NaOD): δ 0.37 (s, 3H), 0.46 (s, 3H), 4.37 (s, 2H), 6.69 (dd, 2H, *J* = 8.8 Hz, 2.4 Hz), 6.91 (d, 2H, *J* = 2.4 Hz), 7.01-7.10 (m, 4H). ^{13}C NMR (75 MHz, CD_3OD+NaOD): δ -0.9, 0.0, 60.5, 85.0, 118.8, 119.1, 122.0, 129.8, 132.5, 136.6, 138.7, 139.4, 147.1, 147.4. HRMS (ESI$^+$): Calcd for $[M+H]^+$, 365.11438, Found, 365.11429 (-0.09 mmu)

24 gGlu-HMRR (gGlu-HM3ThPSiR600)

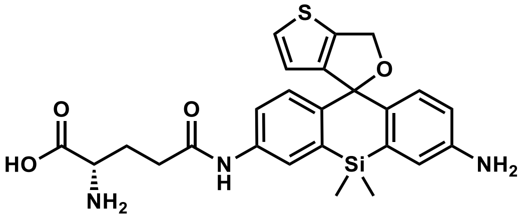

Compound 23 (30 mg, 0.082 mmol), boc-Glu-OtBu (12.4 mg, 0.041 mmol) and N,N-diisopropylethylamine (106 mg, 0.82 mmol) were dissolved in anhydrous DMF (2 mL), and the solution was stirred at r.t.. HATU (15.6 mg, 0.041 mmol) was added, and stirring was continued for 1 hour. The mixture was evaporated and the residue was dissolved in CH_2Cl_2 (5 mL) and trifluoroacetic acid (5 mL). The resulting solution was stirred at 40˚C for 1 hour, and then evaporated. The residue was purified by preparative HPLC under the following conditions: A/B = 80/20 (0 min) to 0/100 (45 min) linear gradient (solvent A: H_2O, 0.1% TFA; solvent B: acetonitrile/H_2O = 80/20, 0.1% TFA). Compound 24 was obtained as a red solid (16 mg, 80%). ^1H NMR (400 MHz, CD_3OD): δ 0.55 (s, 6H), 2.18-2.36 (m, 2H), 2.74-2.79 (m, 2H), 4.09 (t, 1H, *J* = 6.4 Hz), 4.42 (s, 2H), 6.85 (d, 1H, *J* = 8.0 Hz), 6.97 (d, 1H, *J* = 5.2 Hz), 7.21 (d, 1H, *J* = 8.8 Hz), 7.41 (d, 1H, *J* = 8.0 Hz), 7.46 (s, 1H), 7.62 (d, 1H, *J* = 5.2 Hz), 7.72 (dd, 1H, *J* = 8.8 Hz, 2.4 Hz), 8.15 (d, 1H, *J* = 2.4 Hz) HRMS (ESI^+): Calcd for $[M+H]^+$, 494.15698, Found, 494.15708 (+0.10 mmu).

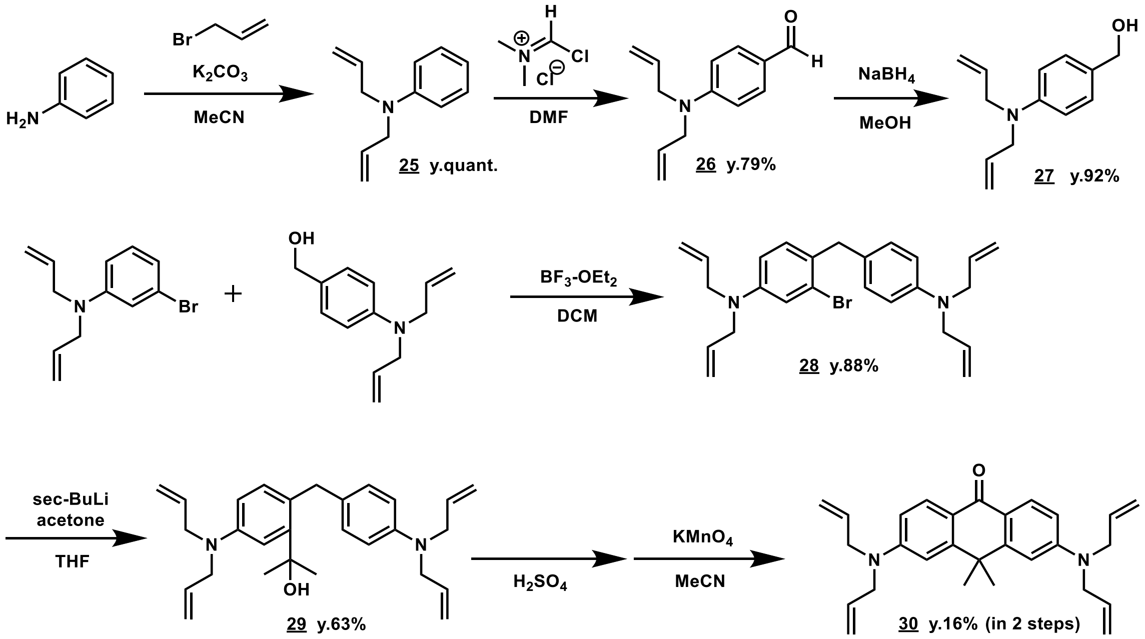

25

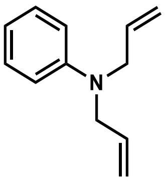

Aniline (9.8 mL, 107.4 mmol), K_2CO_3 (29.7 g, 214.8 mmol) and allyl bromide (20 mL, 236.3 mmol) were added in MeCN (100 mL), and the mixture was refluxed at 100 °C for 16 h. After cooling, the reaction mixture was filtered, extracted with AcOEt and evaporated. The residue was purified by column chromatography (silica gel, hexane/AcOEt = 9/1) to give compound 25 as colorless liquid (20.8 g, quant.). ^{1}H NMR (400 MHz, $CDCl_3$) δ 3.85-3.95 (m, 4H), 5.11-5.19 (m, 4H), 5.80-5.89 (m, 2H), 6.70-6.65 (m, 3H), 7.20-7.15 (m, 2H).

26

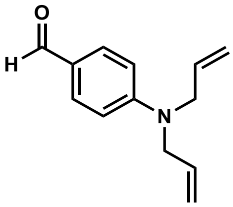

Vilsmeier reagent (7.4 g, 57.7 mmol) was dissolved in anhydrous DMF (40 mL) and the solution was stirred at 0˚C under an Ar atmosphere. Then, compound 25 (10.0 g, 10.9 mL, 57.7 mmol) was added, and stirring was continued at r.t. for 20 hours. The reaction was quenched with saturated $NaHCO_3$ aq. and the mixture was extracted with CH_2Cl_2. The organic solution was dried over Na_2SO_4, filtered, and evaporated. The residue was purified by flash column chromatography (silica gel, *n*-hexane/AcOEt = 9/1 to 2/1) to give compound 26 as a colorless liquid (9.14 g, 79%). ^{1}H NMR (400 MHz, $CDCl_3$): δ 3.99 (d, 4H, *J* = 5.6 Hz), 5.12-5.20 (m, 4H), 5.79-5.87 (m, 2H), 6.69 (d, 2H, *J* = 9.2 Hz), 7.69 (d, 2H, *J* = 9.2 Hz), 9.71 (s, 1H). ^{13}C NMR (100 MHz, $CDCl_3$): δ 52.8, 111.5, 116.8, 125.7, 132.1, 132.3, 153.3, 190.3.

27

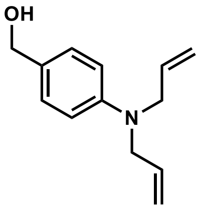

Compound 26 (8000 mg, 39.7 mmol) was dissolved in anhydrous methanol (50 mL) and stirred at 0˚C. Sodium tetrahydroborate (1654 mg, 43.7 mmol) was added and stirring was continued at r.t. for 4 hours. The reaction was quenched with H_2O and the mixture was extracted with CH_2Cl_2. The organic solution was dried over Na_2SO_4, filtered, and evaporated. The residue was purified by flash column chromatography (silica gel, *n*-hexane/AcOEt = 2/1 to 1/1) to give compound 27 as a colorless liquid (7450 mg, 92%). ^{1}H NMR (400 MHz, $CDCl_3$): δ 3.92 (d, 4H, *J* = 4.0 Hz), 4.53 (s, 2H), 5.14-5.19 (m, 4H), 5.80-5.89 (m, 2H), 6.67 (d, 2H, *J* = 9.2 Hz), 7.19 (d, 2H, *J* = 9.2 Hz). ^{13}C NMR (100 MHz, $CDCl_3$): δ 52.9, 65.4, 112.4, 116.1, 128.7, 128.8, 133.9, 148.5. HRMS (ESI$^+$): Calcd for $[M+H]^+$, 204.13884, Found, 204.13520 (-3.64 mmu).

28

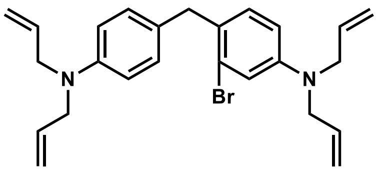

Compound 16 (2522 mg, 10.0 mmol) and compound 27 (2030 mg, 10.0 mmol) were dissolved in anhydrous CH_2Cl_2 (20 mL) and the solution was stirred at 0˚C. Boron trifluoride ethyl ether complex (2.5 mL, 20.0 mmol) was added, and stirring was continued at r.t. for 22 hours. The reaction was quenched with saturated $NaHCO_3$ aq. and the mixture was extracted with CH_2Cl_2. The organic solution was dried over Na_2SO_4, filtered, and evaporated. The residue was purified by flash column chromatography (silica gel, *n*-hexane/AcOEt = 10/0 to 8/2) to give compound 28 as a colorless liquid (3870 mg, 88%). ^{1}H NMR (400 MHz, $CDCl_3$): δ 3.85-3.90 (m, 10H), 5.12-5.19 (m, 8H), 5.77-5.90 (m, 4H), 6.55 (dd, 1H, *J* = 8.4 Hz, 2.8 Hz), 6.63 (d, 2H, *J* = 8.4 Hz), 6.87 (d, 1H, *J* = 2.8 Hz), 6.93 (d, 1H, *J* = 8.4 Hz), 7.02 (d, 2H, *J* = 8.4 Hz). ^{13}C NMR (100 MHz, $CDCl_3$): δ 39.7, 52.8, 52.9, 111.8, 112.5, 116.0, 116.1, 116.3, 125.5, 128.4, 128.6, 129.6, 131.1, 133.6, 134.4, 147.1, 148.1. HRMS (ESI$^+$): Calcd for $[M+H]^+$, 437.15924, 439.15719, Found, 437.16055, 439.15909 (+1.32 mmu, +1.90 mmu)

29

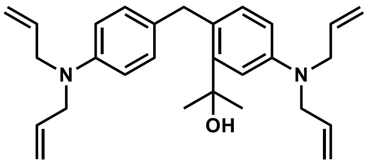

To a flame-dried flask flushed with Ar, compound 28 (1800 mg, 4.1 mmol) and anhydrous THF (15 mL) were added. The mixture was cooled to -78˚C and then 1 M *sec*-BuLi (4.1 mL, 4.1 mmol) was added to it. Acetone (0.6 mL, 8.2 mmol) was further added, and the mixture was stirred at r.t. for 3 hours. The reaction was quenched with H_2O and the mixture was extracted with CH_2Cl_2 from saturated $NaHCO_3$ aq.. The organic solution was dried over Na_2SO_4, filtered, and evaporated. The residue was purified by flash column chromatography (silica gel, *n*-hexane/AcOEt = 10/0 to 8/2) to give compound 29 as a colorless solid (1073 mg, 63%).^{1}H NMR (400 MHz, $CDCl_3$): δ 1.60 (s, 6H), 1.76 (s, 1H), 3.87-3.91 (m, 8H), 4.16 (s, 2H), 5.11-5.22 (m, 8H), 5.79-5.92 (m, 4H), 6.56 (d, 1H, *J* = 8.0 Hz), 6.61 (d, 2H, *J* = 7.2 Hz), 6.82 (s, 1H), 6.93-6.97 (m, 3H). ^{13}C NMR (75 MHz, $CDCl_3$): δ 31.8, 38.0, 53.0, 53.2, 74.3, 110.2, 111.3, 112.6, 116.0, 116.2, 126.5, 129.4, 130.9, 134.0, 134.4, 134.6, 146.6, 146.9, 146.9.

30

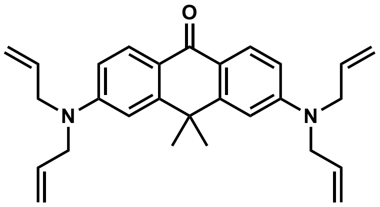

Compound 29 (8900 mg, 21.4 mmol) was dissolved in 95% H_2SO_4 (10 mL) and the solution was stirred at 0˚C for 10 minutes. The reaction was quenched with saturated Na_2CO_3 aq. and the mixture was extracted with CH_2Cl_2. The organic solution was dried over Na_2SO_4, filtered, and evaporated. The residue was dissolved in acetonitrile (120 mL) and the solution was stirred at 0˚C. $KMnO_4$ (10128 mg, 64.1 mmol) was added portionwise, then the mixture was stirred at r.t. for 2 hours, and the reaction was quenched with methanol. The mixture was filtered through Celite, and the filtrate was evaporated. The residue was purified by flash column chromatography (silica gel, CH_2Cl_2/methanol = 100/0 to 97/3) to give compound 30 as a light yellow solid (1420 mg, 16%). ^{1}H NMR (400 MHz, $CDCl_3$): δ 1.63 (s, 6H), 4.02 (d, 8H, *J* = 2.8 Hz), 5.20-5.23 (m, 8H), 5.84-5.93 (m, 4H), 6.72 (dd, 2H, *J* = 2.0 Hz, 8.8 Hz), 6.76 (d, 2H, *J* = 2.0 Hz), 8.20 (d, 2H, *J* = 8.8 Hz). ^{13}C NMR (100 MHz, $CDCl_3$): δ 33.6, 38.1, 53.0, 108.5, 111.1, 116.6, 120.3, 129.2, 133.3, 151.8, 152.3, 181.1. HRMS (ESI$^+$): Calcd for $[M+H]^+$, 413.25929, Found, 413.25696(-2.23mmu)

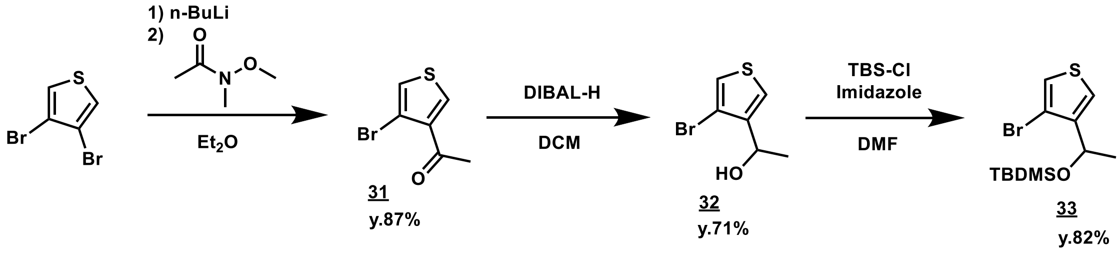

31

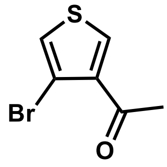

To a flame-dried flask flushed with Ar, 3,4-Dibromothiophene (3500 mg, 14.5 mmol) and anhydrous Et_2O (30 mL) were added. The mixture was cooled to -78˚C and then 1 M *n*-BuLi (10 mL, 15.9 mmol) was added to it and stirred 15 min. *N*-Methoxy-*N*-methylacetamide (1.8 mL, 17.4 mmol) in Et_2O (2 mL) was further added and stirred 30 min, then the mixture was warmed to r.t. and stirred for 30 min. The reaction was quenched with H_2O and the mixture was extracted with AcOEt from saturated NH_4Cl aq.. The organic solution was dried over Na_2SO_4, filtered, and evaporated. The residue was purified by flash column chromatography (silica gel, *n*-hexane/AcOEt = 10/0 to 7/3) to give compound 31 as a colorless solid (2573 mg, 87%).^{1}H NMR (400 MHz, $CDCl_3$): δ 2.60 (s, 3 H), 7.31 (d, 1H, J = 3.6 Hz), 8.01 (d, 1H, J = 3.6 Hz).

32

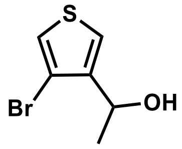

Compound 31 (1000 mg, 4.9 mmol) was dissolved in anhydrous THF (20 mL), and the solution was stirred at 0˚C. Sodium tetrahydroborate (278 mg, 7.4 mmol) was added and stirring was continued at r.t. for 22 hours. The reaction was quenched with 1 N HCl aq. and the mixture was extracted with CH_2Cl_2. The organic solution was dried over Na_2SO_4, filtered, and evaporated. The residue was purified by flash column chromatography (silica gel, *n*-hexane/AcOEt = 9/1 to 7/3) to give compound 32 as a colorless liquid (714 mg, 71%). ^{1}H NMR (400 MHz, $CDCl_3$): δ 1.49 (d, 3H, J = 6.8 Hz), 2.43 (d, 1H, J = 4.4 Hz), 4.91-4.97 (m, 1H), 7.23 (d, 1H, J = 3.6 Hz), 7.27 (d, 1H, J = 3.6 Hz). ^{13}C NMR (100 MHz, $CDCl_3$): δ 23.4, 66.0, 109.9, 121.3, 123.7, 145.2.

33

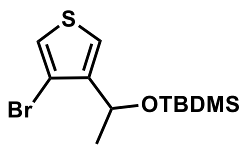

Compound 32 (1077 mg, 5.23 mmol), tert-butyldimethylchlorosilane (2366 mg, 15.7 mmol) and imidazole (2136 mg, 31.4 mmol) were dissolved in anhydrous DMF (12 mL). The solution was stirred at r.t. under an Ar atmosphere for 4 hours, then extracted with *n*-hexane from brine. The organic solution was dried over Na_2SO_4, filtered, and evaporated. The residue was purified by flash column chromatography (silica gel, *n*-hexane/AcOEt = 10/0 to 9/1) to give compound 33 as a colorless liquid (1384 mg, 82%). ^{1}H NMR (400 MHz, $CDCl_3$): δ 0.01 (s, 3H), 0.06 (s, 3H), 0.90 (s, 9H), 1.41 (d, 3H, *J* = 6.0 Hz), 4.91 (q, 1 H, *J* = 6.0 Hz), 7.20 (d, 1H, *J* = 3.6 Hz), 7.26 (d, 1H, *J* = 3.6 Hz). ^{13}C NMR (100 MHz, $CDCl_3$): δ -4.86, -4.82, 18.3, 25.6, 25.9, 67.5, 108.9, 121.2, 123.1, 146.5.

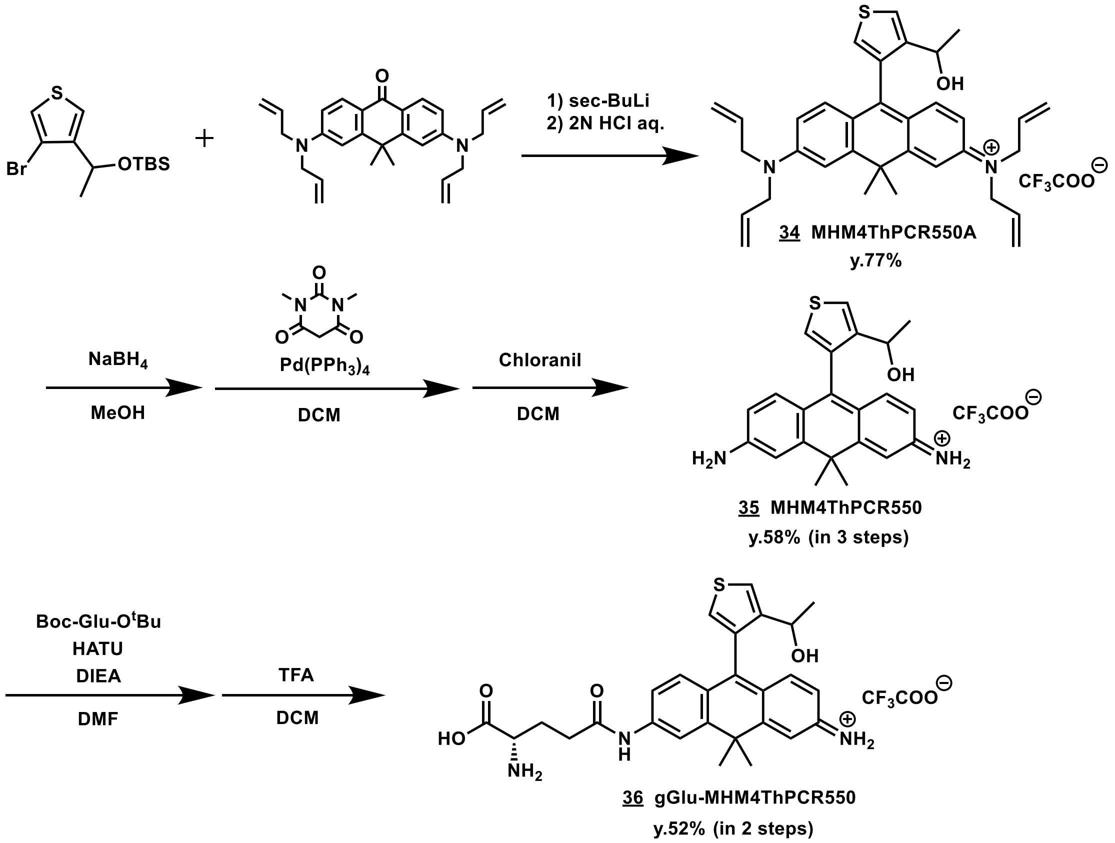

34

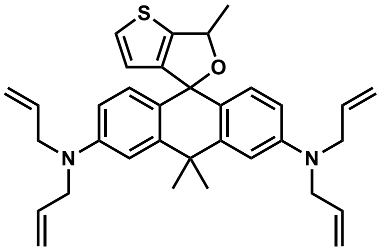

To a flame-dried flask flushed with Ar, compound 33 (546 mg, 1.7 mmol) and anhydrous THF (12 mL) were added. The mixture was cooled to -85˚C and then 1 M *sec*-BuLi (1.6 mL, 1.7 mmol) was added to it. Compound 30 (140 mg, 0.34 mmol) in anhydrous THF (4 mL) was further added. The mixture was stirred at r.t. for 1 hour, then the reaction was quenched with 2 N HCl aq., and the mixture was extracted with CH_2Cl_2 from saturated $NaHCO_3$ aq.. The organic solution was dried over Na_2SO_4, filtered, and evaporated. The residue was purified by preparative HPLC under the following conditions: A/B = 80/20 (0 min) to 0/100 (30 min) linear gradient (solvent A: H_2O, 0.1% TFA; solvent B: acetonitrile/H_2O = 80/20, 0.1% TFA). Compound 34 was obtained as a dark violet solid (137 mg, 77%). ^{1}H NMR (400 MHz, CD_3OD+NaOD): δ 1.23 (d, 3H, *J* = 6.0 Hz), 1.69 (s, 3H), 1.74 (s, 3H), 4.28-4.32 (m, 8H), 4.46 (q, 1H, *J* = 6.0 Hz), 5.26 (d, 4H, *J* = 17.6 Hz), 5.28 (d, 4H, *J* = 10.4 Hz), 5.89-5.97 (m, 4H). ^{13}C NMR (100 MHz, CD_3OD+NaOD): δ 23.3, 32.0, 33.7, 41.6, 53.5, 64.5, 111.7, 111.7, 113.4, 113.4, 116.6, 121.3, 121.4, 122.1, 126.8, 131.4, 134.2, 137.7, 138.0, 147.4, 156.4, 156.4, 157.2, 161.8. HRMS (ESI$^+$): Calcd for $[M+H]^+$, 523.27831, Found, 523.27729 (-1.02 mmu)

35 HMRY (MHM4ThPCR550)

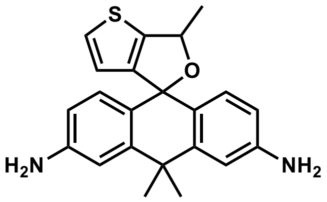

Compound 34 (125 mg, 0.24 mmol) was dissolved in methanol (20 mL), and the solution was stirred at 0˚C. Sodium tetrahydroborate (18 mg, 0.48 mmol) was added and stirring was continued at r.t. for 15 minutes. The reaction was quenched with saturated $NaHCO_3$ aq. and the mixture was extracted with CH_2Cl_2. The organic solution was dried over Na_2SO_4, filtered, and evaporated. The residue was dissolved in dehydrated CH_2Cl_2 (20 mL), and 1,3-dimethylbarbituric acid (186 mg, 1.19 mmol) and $Pd(PPh_3)_4$ (58 mg, 0.05 mmol) were added. The resulting solution was stirred at 35˚C under an Ar atmosphere for 14 hours. Then chloranil (118 mg, 0.48 mmol) was added, and stirring was continued at r.t. for 30 minutes. The mixture was extracted with CH_2Cl_2 from 2 N NaOH aq.. The organic solution was dried over Na_2SO_4, filtered, and evaporated. The residue was purified by preparative HPLC under the following conditions: A/B = 80/20 (0 min) to 0/100 (60 min) linear gradient (solvent A: H_2O, 0.1% TFA; solvent B: acetonitrile/H_2O = 80/20, 0.1% TFA). Compound 35 was obtained as a violet solid (51 mg, 58%). ^{1}H NMR (400 MHz, CD_3OD): δ 1.23 (d, 3H, *J* = 6.4 Hz), 1.66 (s, 3H), 1.71 (s, 3H), 4.46 (q, 1H, *J* = 6.4 Hz), 6.62 (dd, 2H, *J* = 9.2 Hz, 3.2 Hz), 7.12 (d, 1H, *J* = 3.2 Hz), 7.13 (d, 1H, *J* = 3.2 Hz), 7.16 (d, 1H, *J* = 9.2 Hz), 7.21 (d, 1H, *J* = 9.2 Hz), 7.42 (d, 1H, *J* = 3.2 Hz), 7.63 (d, 1H, *J* = 3.2 Hz). ^{13}C NMR (100 MHz, CD_3OD): δ 23.4, 31.5, 33.4, 41.0, 64.6, 112.6, 112.7, 114.6, 114.6, 120.7, 120.9, 121.9, 126.5, 134.4, 138.5, 138.8, 147.4, 157.9, 159.3, 159.4, 161.4. HRMS (ESI$^+$): Calcd for $[M+H]^+$, 363.15311, Found, 363.15147 (-1.64 mmu)

36 gGlu-HMRY (gGlu-MHM4ThPCR550)

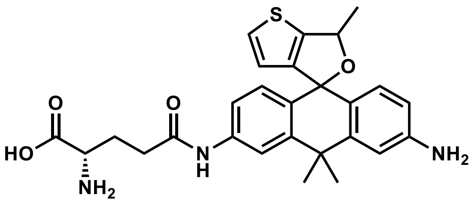

Compound 35 (31 mg, 0.085 mmol), boc-Glu-OtBu (13 mg, 0.043 mmol) and N,N-diisopropylethylamine (110 mg, 0.85 mmol) were dissolved in anhydrous DMF (2 mL), and the solution was stirred at r.t.. HATU (16.2 mg, 0.043 mmol) was added, and stirring was continued for 2 hours, then the mixture was evaporated. The residue was dissolved in CH_2Cl_2 (5 mL) and trifluoroacetic acid (5 mL), and the resulting solution was stirred at 40˚C for 1 hour, and evaporated. The residue was purified by preparative HPLC under the following conditions: A/B = 80/20 (0 min) to 0/100 (45 min) linear gradient (solvent A: H_2O, 0.1% TFA; solvent B: acetonitrile/H_2O = 80/20, 0.1% TFA). Compound 36 was obtained as an orange solid (11 mg, 52%). ^{1}H NMR (400 MHz, CD_3OD): δ 1.24-1.30 (m, 3H), 1.71 (s, 3H), 1.77 (s, 3H), 2.20-2.32 (m, 2H), 2.75 (t, 2H, *J* = 7.2 Hz), 4.05 (t, 2H, *J* = 7.2 Hz), 4.42-4.50 (m, 1H), 6.83 (t, 1H, *J* = 8.4 Hz), 7.20-7.34 (m, 2H), 7.42-7.52 (m, 2H), 7.59 (d, 1H, *J* = 8.4 Hz), 7.66 (t, 1H, *J* = 3.2Hz), 8.26 (s, 1H). ^{13}C NMR (100 MHz, CD_3OD): δ 22.9, 23.3, 25.4, 31.1, 31.1, 32.2, 33.1, 41.3, 52.4, 64.4, 64.6, 115.2, 115.3, 116.9, 117.0, 117.8, 118.0, 118.1, 122.4, 122.6, 124.5, 124.6, 125.8, 125.9, 127.3, 127.3, 133.7, 134.0, 134.4, 134.8, 141.8, 142.2, 145.4, 145.4, 147.1, 147.5, 152.6, 160.8, 161.1, 161.8, 163.4, 163.5, 170.6, 171.9. HRMS (ESI$^+$): Calcd for $[M+H]^+$, 492.19570, Found, 492.19380 (-1.90 mmu)

Supplementary Figures and Tables

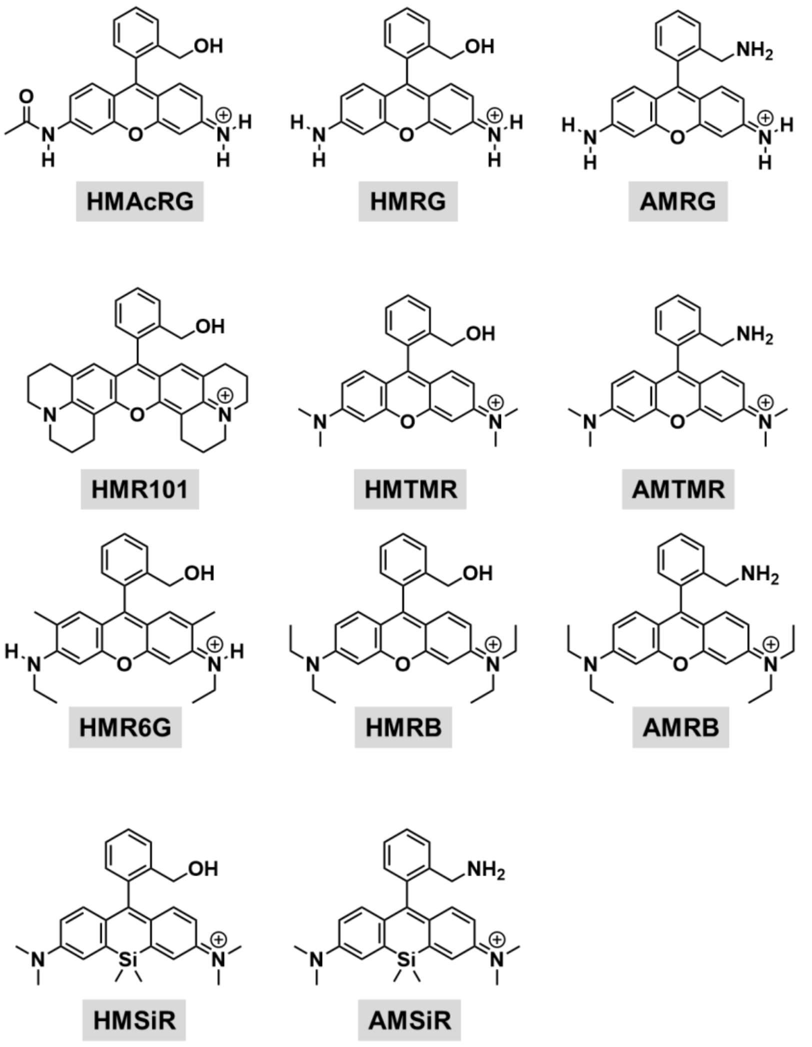

Supplementary Figure 1 Structures and names of typical HMR derivatives.

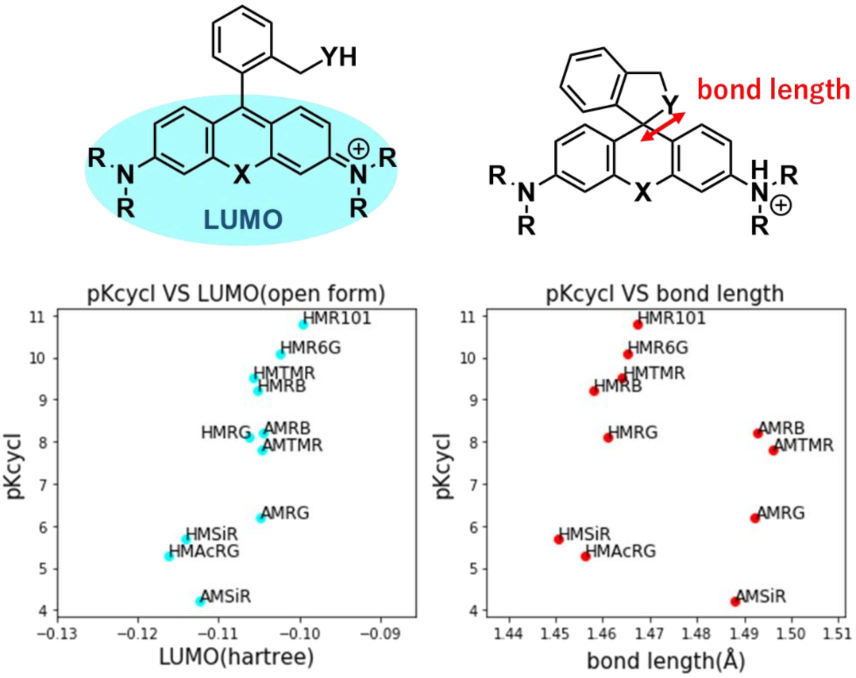

Supplementary Figure 2 Correlation between pK_{cycl} and LUMO energy or bond length of the spiro ring of HMR derivatives (HMRG, HMTMR, HMRB, HMSiR, AMRG, AMTMR, AMRB, AMSiR, HMR101, HMR6G, HMAcRG).

$$pK_{cycl} = log\frac{K_A - 1}{K_{aNH} - K_{aOH}K_A}$$

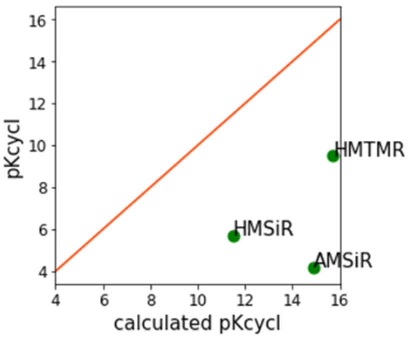

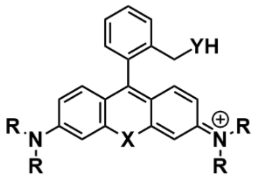

	Measured pK_{cycl}	Calculated pK_{cycl}	R	X	Y
**HMRG**	**8.1**	**N/A※**	H	O	O
**AMRG**	**6.2**	**N/A※**	H	O	NH
**HMTMR**	**9.5**	**15.7**	Me	O	O
**AMTMR**	**7.8**	**N/A※**	Me	O	NH
**HMRB**	**9.2**	**N/A※**	Et	O	O
**AMRB**	**8.2**	**N/A※**	Et	O	NH
**HMSiR**	**5.7**	**11.5**	Me	$SiMe_2$	O
**AMSiR**	**4.2**	**14.9**	Me	$SiMe_2$	NH

Supplementary Table 1 Correlation between measured and calculated pK_{cycl} of HMR derivatives (HMRG, HMTMR, HMRB, HMSiR, AMRG, AMTMR, AMRB, AMSiR) without first-shell water molecules. *N/A: K_A is out of domain of the function.

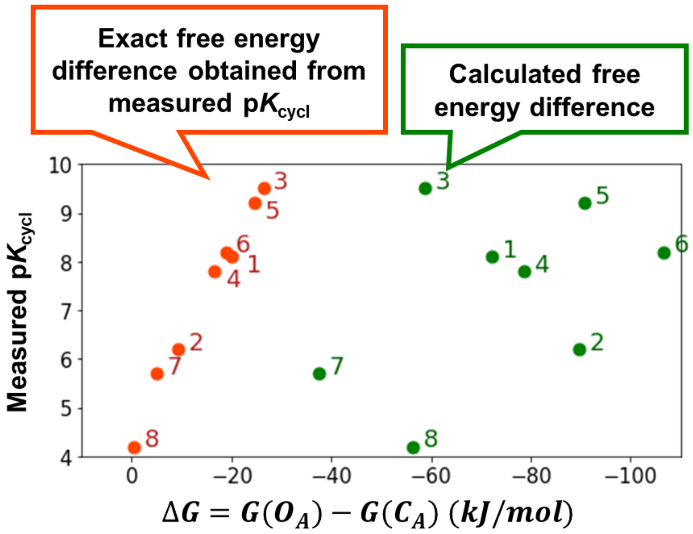

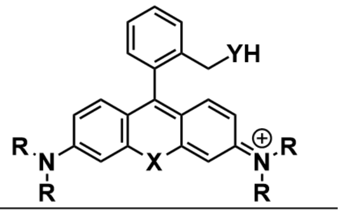

		R	X	Y
1	**HMRG**	H	O	O
2	**AMRG**	H	O	NH
3	**HMTMR**	Me	O	O
4	**AMTMR**	Me	O	NH
5	**HMRB**	Et	O	O
6	**AMRB**	Et	O	NH
7	**HMSiR**	Me	$SiMe_2$	O
8	**AMSiR**	Me	$SiMe_2$	NH

Supplementary Figure 3 Correlation between pK_{cycl} and the free energy difference between the open and closed forms of HMR derivatives (HMRG, HMTMR, HMRB, HMSiR, AMRG, AMTMR, AMRB, AMSiR) without first-shell water molecules.

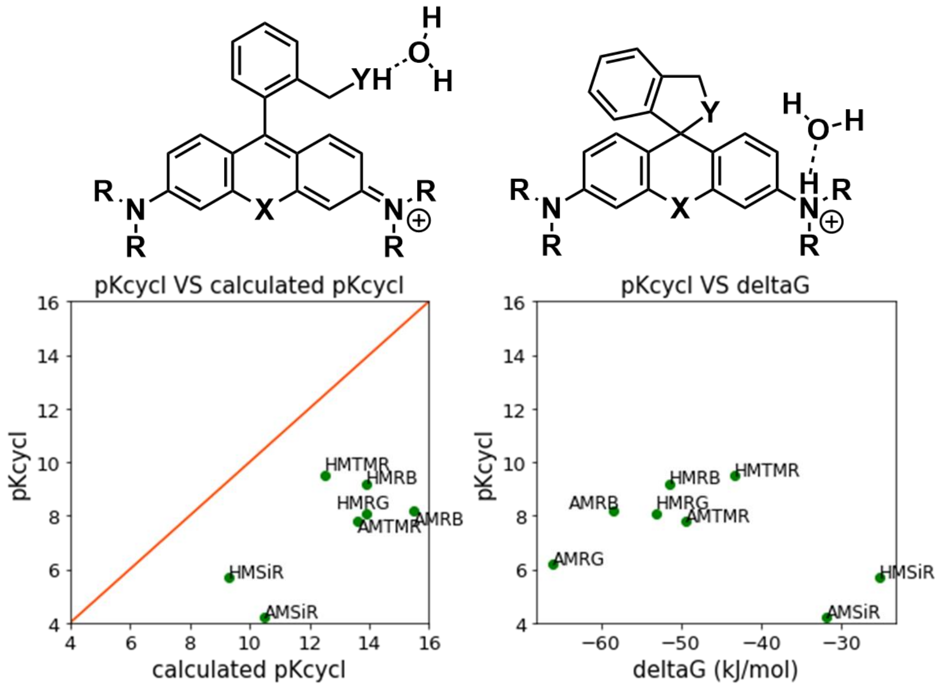

Supplementary Figure 4 Calculation of ΔG and pK_{cycl} (with a first-shell water molecule at the amino group (closed form) and HM group (open form)).

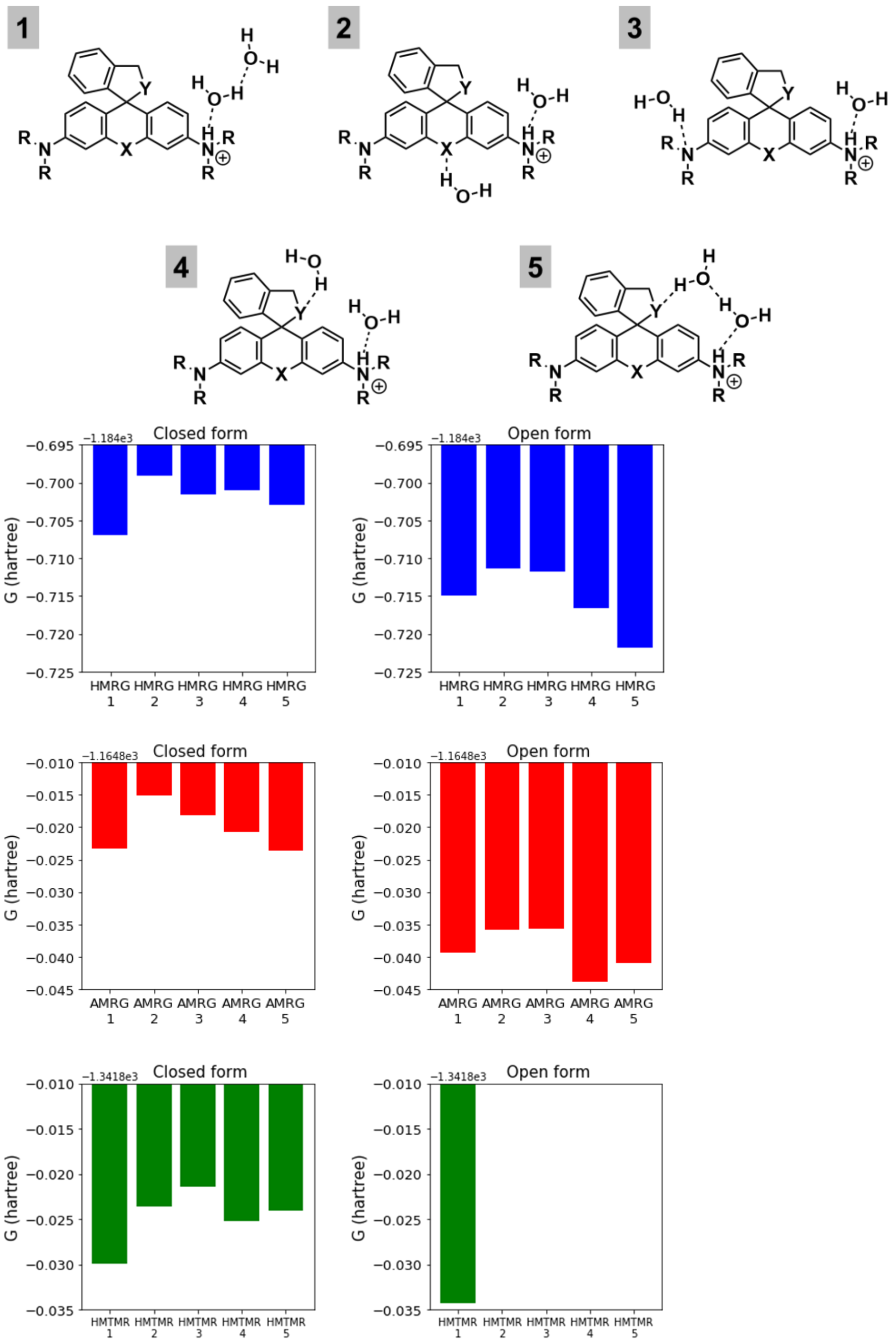

Supplementary Figure 5 Calculated free energy values of HMR derivatives (HMRG, AMRG, HMTMR) with two first-shell water molecules. Structures 2-5 of HMTMR were not obtained in open form.

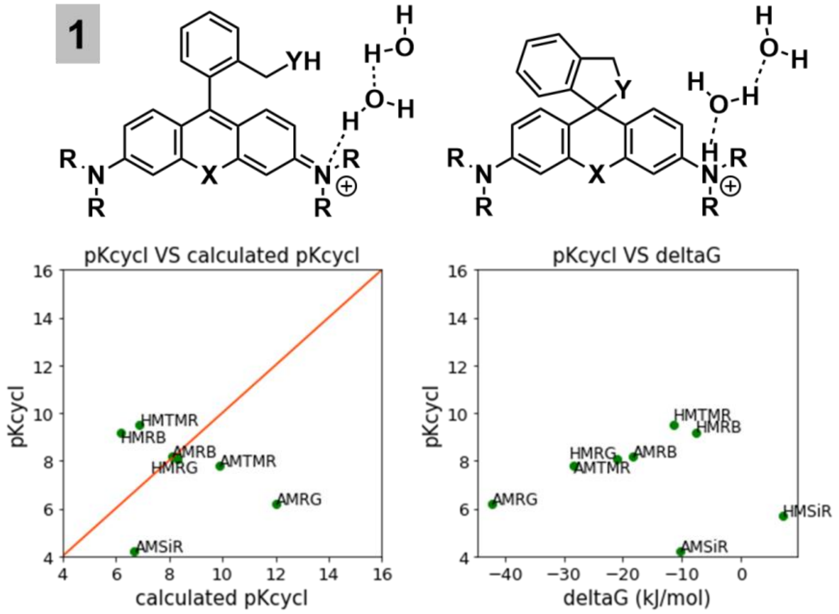

Supplementary Figure 6 Calculation of ΔG and pK_{cycl} (with two first-shell water molecules, structure 1).

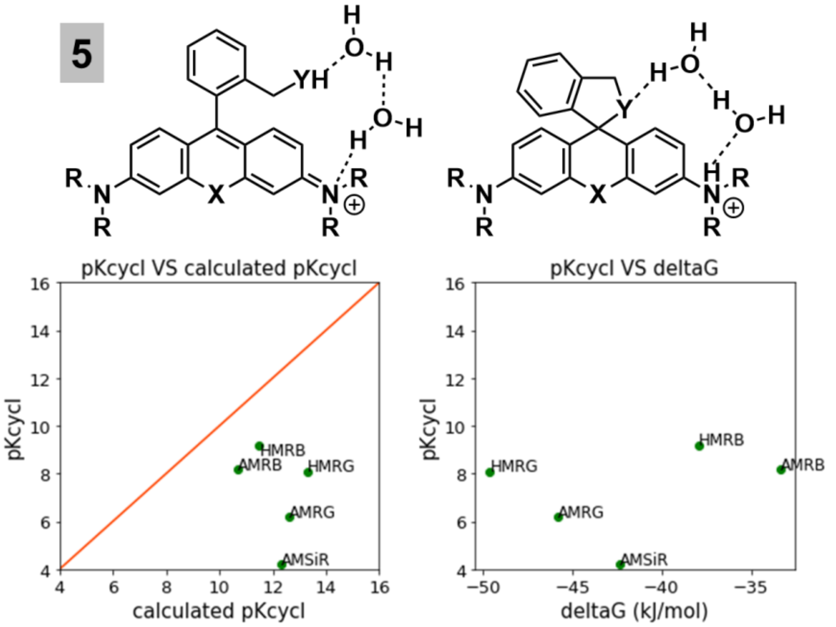

Supplementary Figure 7 Calculation of ΔG and pK_{cycl} (with two first-shell water molecules, structure 5).

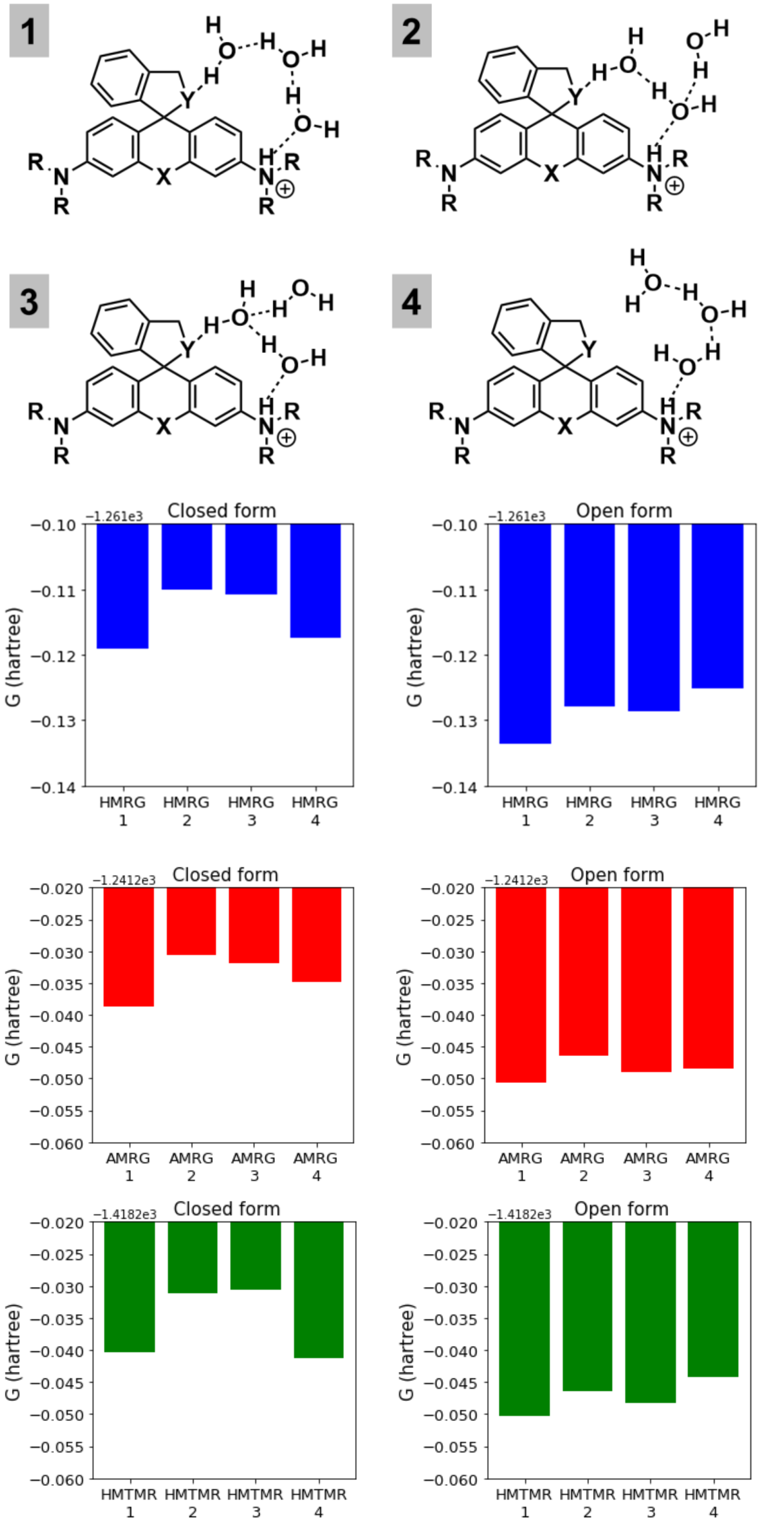

Supplementary Figure 8 Calculated free energy values of HMR derivatives (HMRG, AMRG, HMTMR) with three first-shell water molecules.

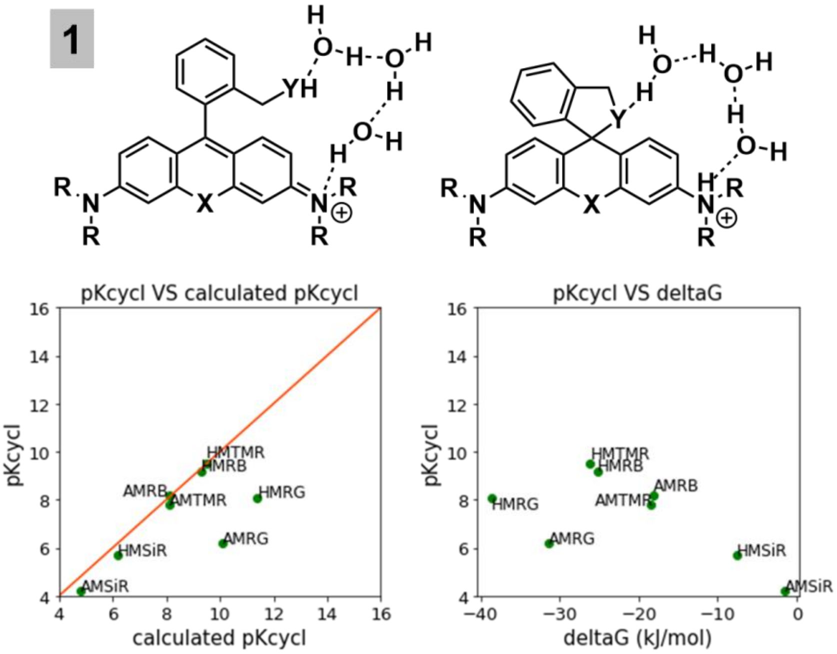

Supplementary Figure 9 Calculation of ΔG and pK_{cycl} (with three first-shell water molecules, structure 1).

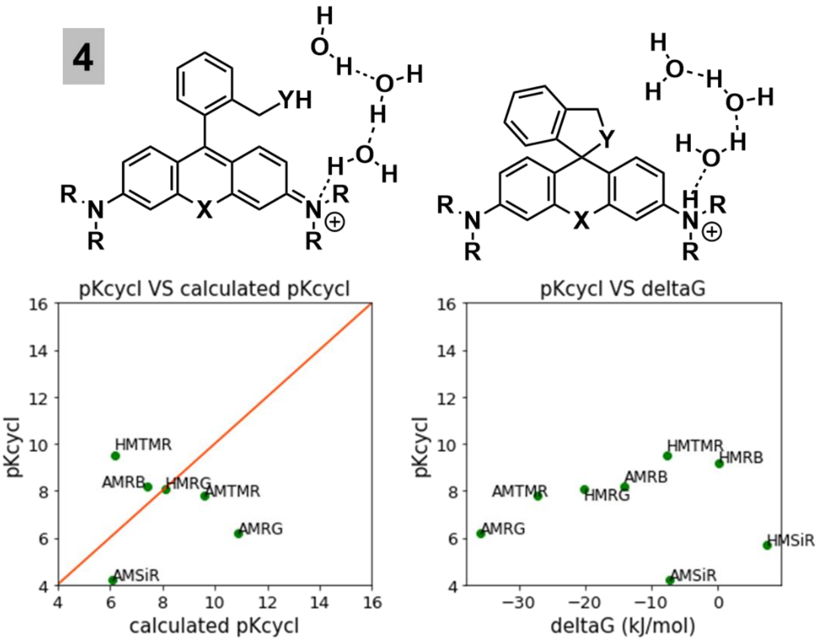

Supplementary Figure 10 Calculation of ΔG and pK_{cycl} (with three first-shell water molecules, structure 4).

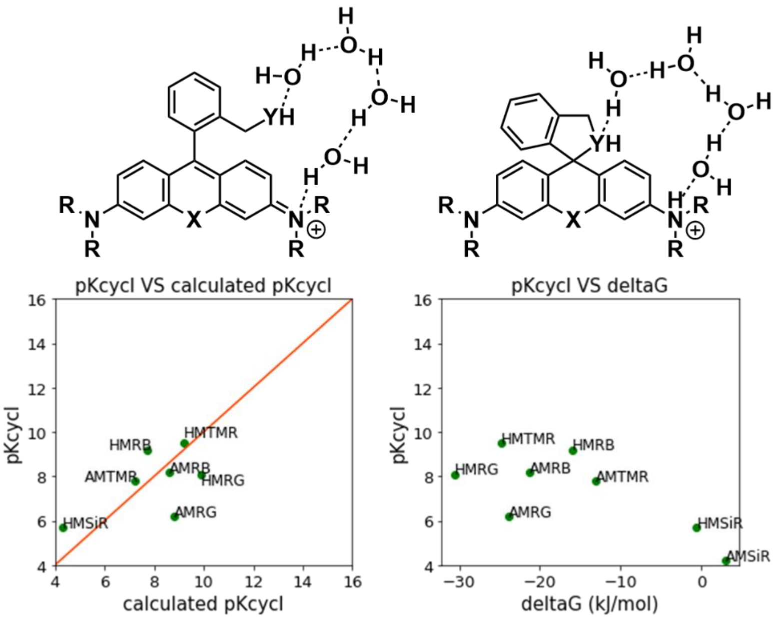

Supplementary Figure 11 Calculation of ΔG and pK_{cycl} (with four first-shell water molecules, bridge structure).

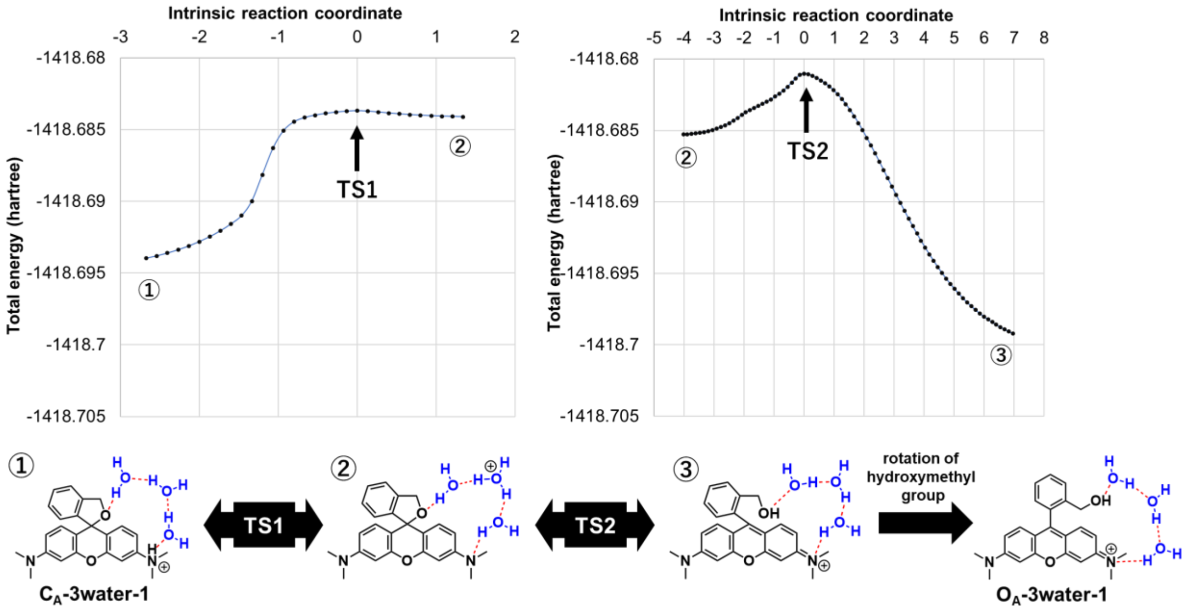

Supplementary Figure 12 IRC calculation of transition states with a bridge of 3 water molecules (HMTMR).

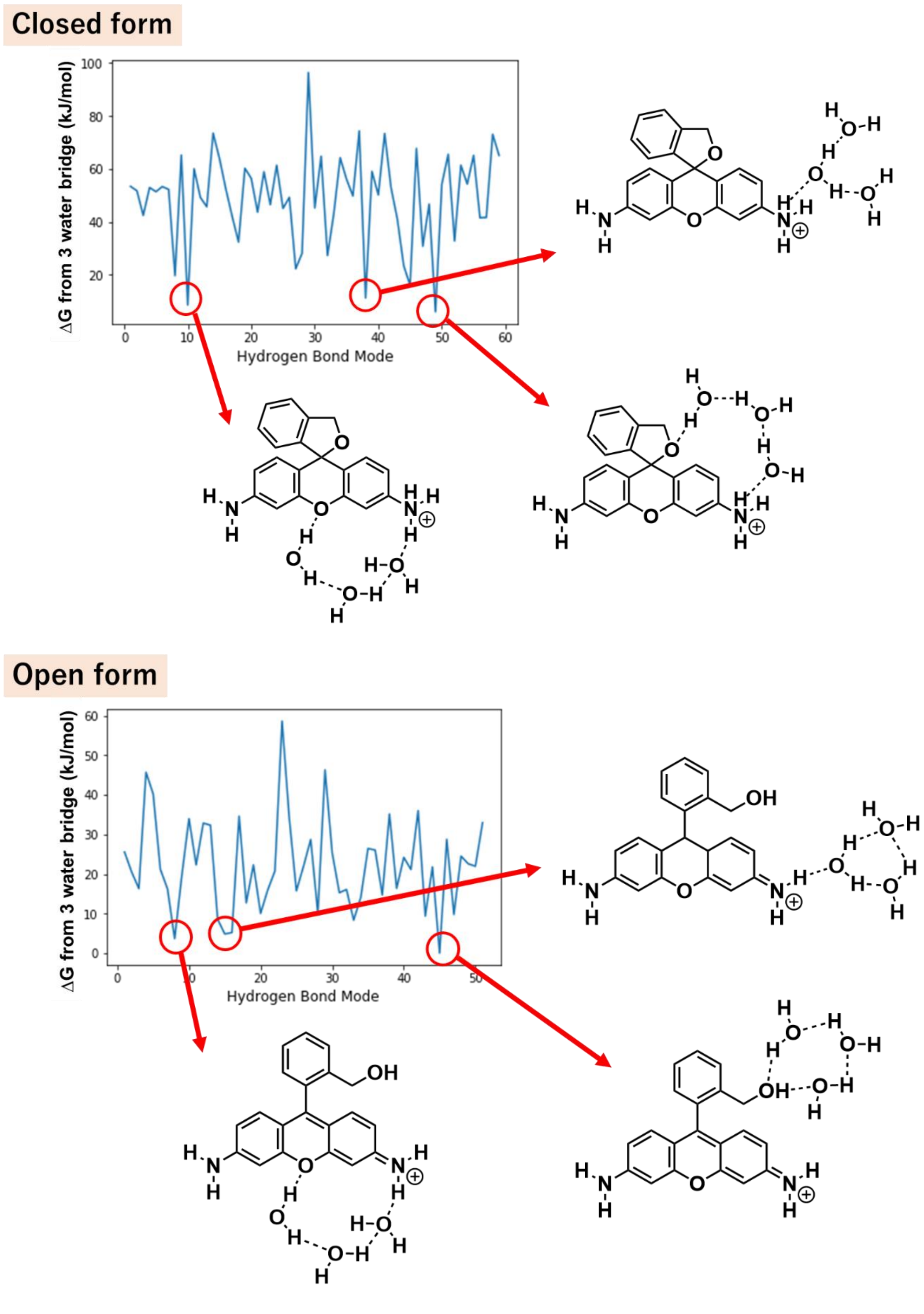

Supplementary Figure 13 Randomized hydrogen bond search (HMRG with 3 water molecules).

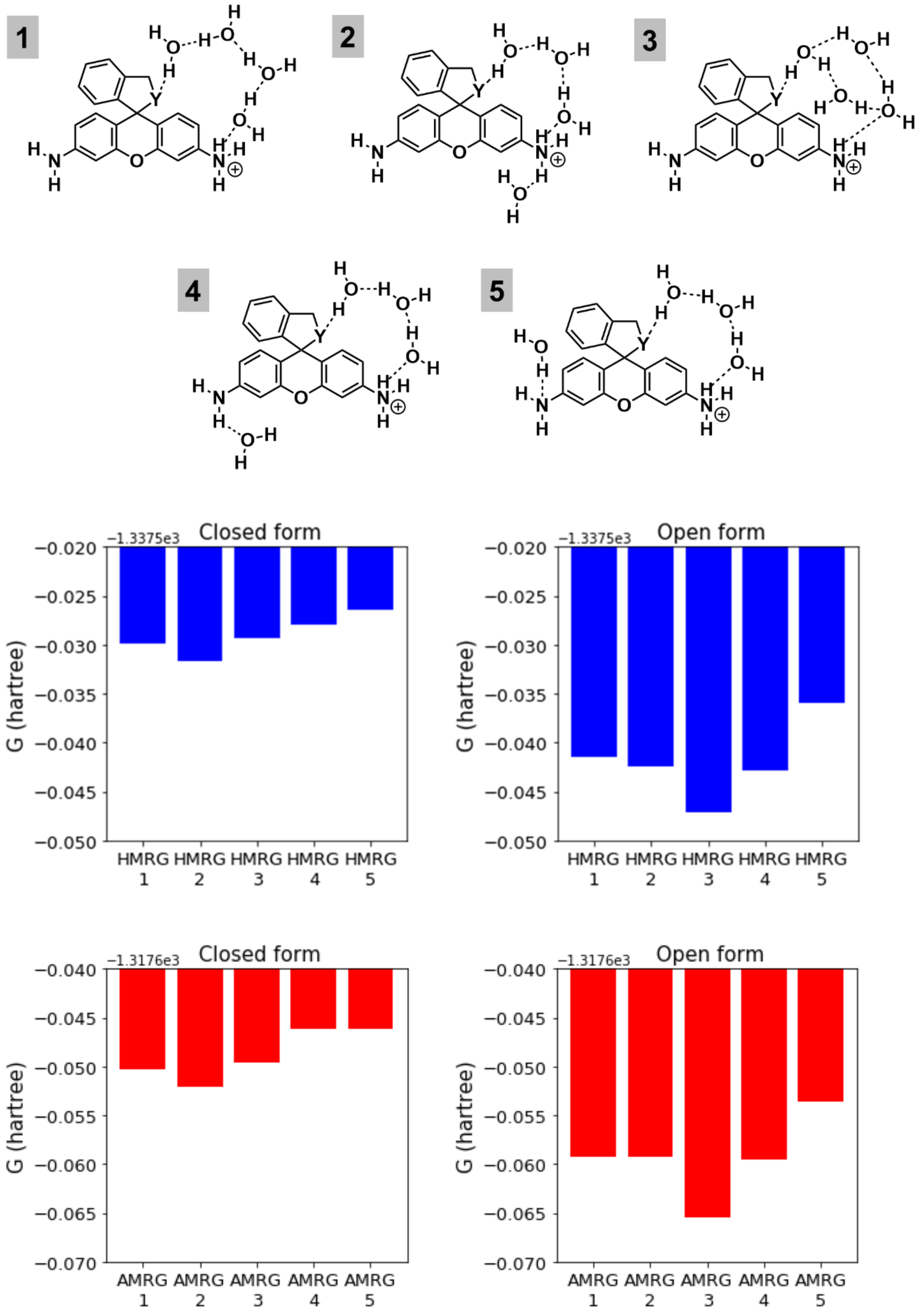

Supplementary Figure 14 Calculated free energy values of HMR derivatives (HMRG, AMRG) with four first-shell water molecules.

	Measured pK_{cycl}	Calculated pK_{cycl} water ①	Calculated pK_{cycl} water ①②	Calculated pK_{cycl} water ①②③
HMRG	8.1	11.3	7.9	-
AMRG	6.2	10.1	6.2	-
HMAcRG	5.3	6.4	< 4	5.8

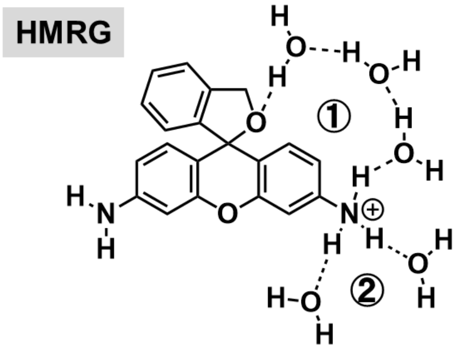

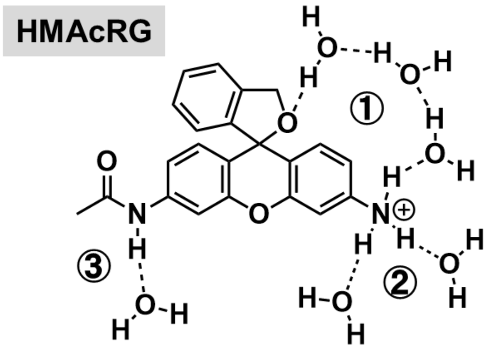

Supplementary Table 2 Calculated pK_{cycl} values of HMRG, AMRG, HMAcRG with first-shell water molecules (3 water bridge and additional molecules).

	Measured pK_{cycl}	Calculated pK_{cycl} No correction	Calculated pK_{cycl} GD3	Calculated pK_{cycl} GD3BJ
HMRG	8.1	7.9	7.0	7.9
AMRG	6.2	6.2	4.6	< 4
HMTMR	9.5	9.5	8.8	9.9
AMTMR	7.8	8.1	6.4	6.1
HMRB	9.2	9.3	7.8	7.2
AMRB	8.2	8.1	4.2	6.9
HMSiR	5.7	6.2	-	5.7
AMSiR	4.2	4.8	< 4	< 4
HMR101	10.8	10.8	10.9	12.1
HMR6G	10.1	10.9	10.5	11.1

Supplementary Table 3 Calculated pK_{cycl} values of HMR derivatives (HMRG, HMTMR, HMRB, HMSiR, AMRG, AMTMR, AMRB, AMSiR, HMR101, HMR6G) including Grimme's correction[1].

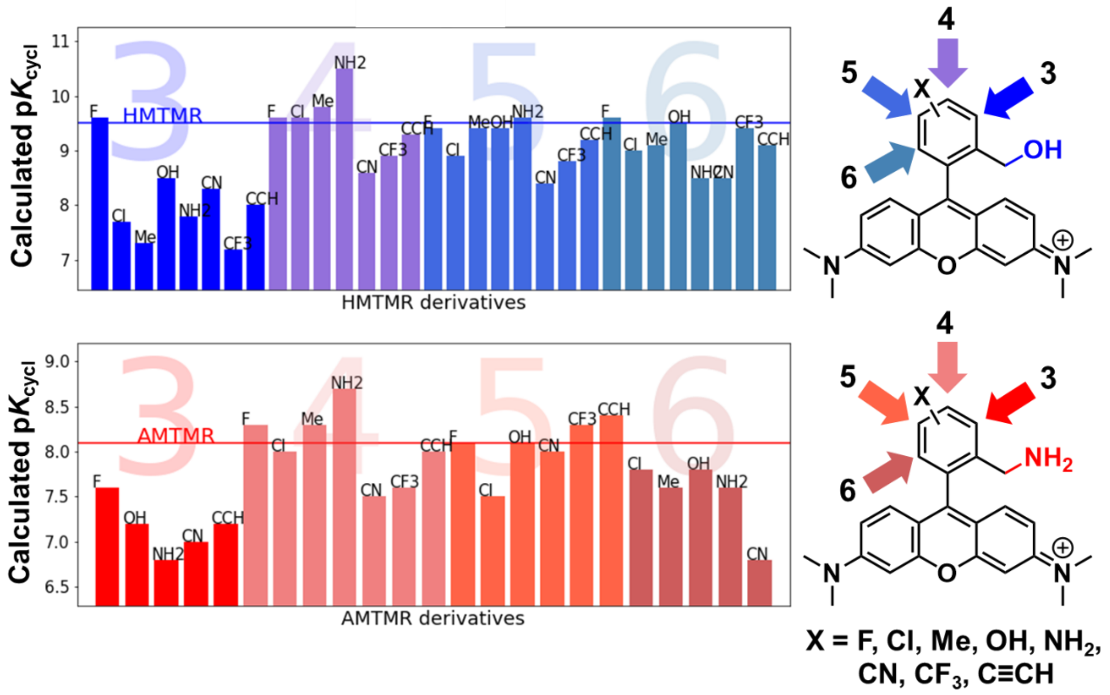

Supplementary Figure 15 Calculated pK_{cycl} values of H(A)MR derivatives substituted with various groups on the benzene moiety.

		Calculated pK_{cycl}		
	Measured pK_{cycl}	No correction	GD3	GD3BJ
**3FHMRG**	8.0	8.1	8.0	6.6
**3CF3HMRG**	5.3	5.3	5.6	4.0
**3MHMRG**	6.6	6.1	5.8	4.9
**5MHMRG**	8.2	7.9	7.9	7.2

Supplementary Table 4 Calculated pK_{cycl} values of HMR derivatives (3FHMRG, 3CF3HMRG, 3MHMRG, 5MHMRG) including Grimme's correction[1].

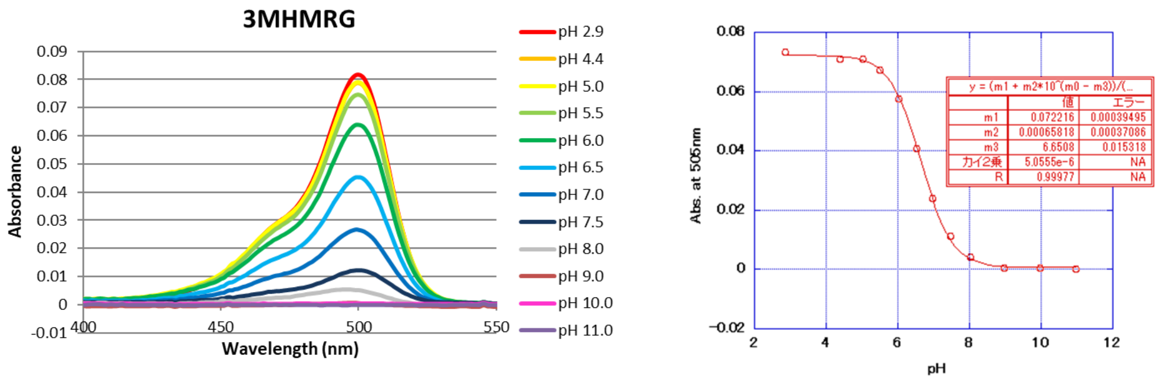

Supplementary Figure 16 Absorption spectra of 1 μM 3MHMRG, and correlation between absorbance at 505 nm and pH in 0.1 M NaPi buffer containing 0.1% DMSO as a cosolvent.

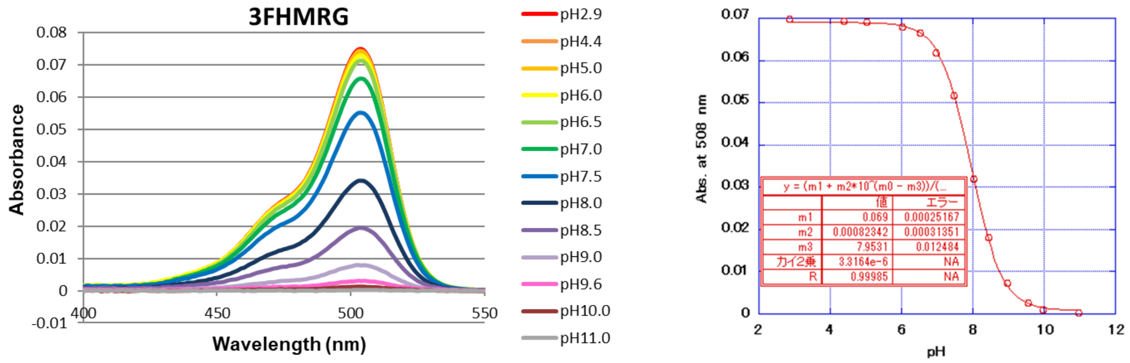

Supplementary Figure 17 Absorption spectra of 1 μM 3FHMRG, and correlation between absorbance at 508 nm and pH in 0.1 M NaPi buffer containing 0.1% DMSO as a cosolvent.

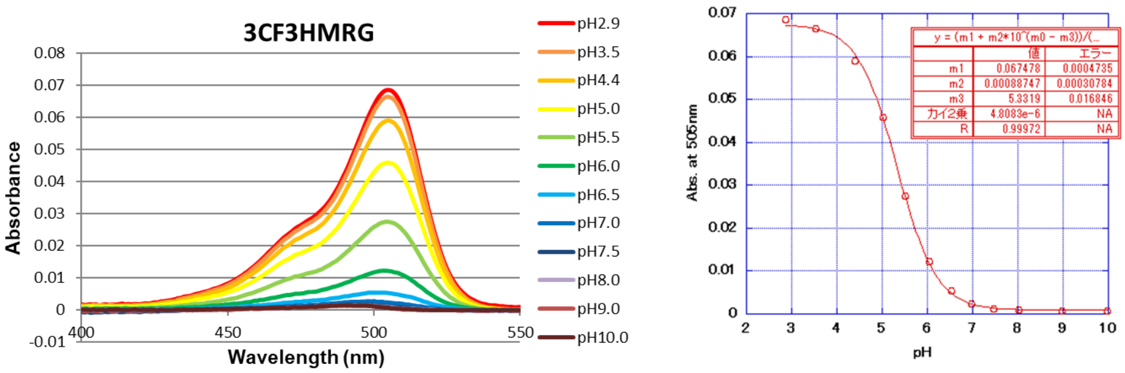

Supplementary Figure 18 Absorption spectra of 1 μM 3CF3HMRG, and correlation between absorbance at 505 nm and pH in 0.1 M NaPi buffer containing 0.1% DMSO as a cosolvent.

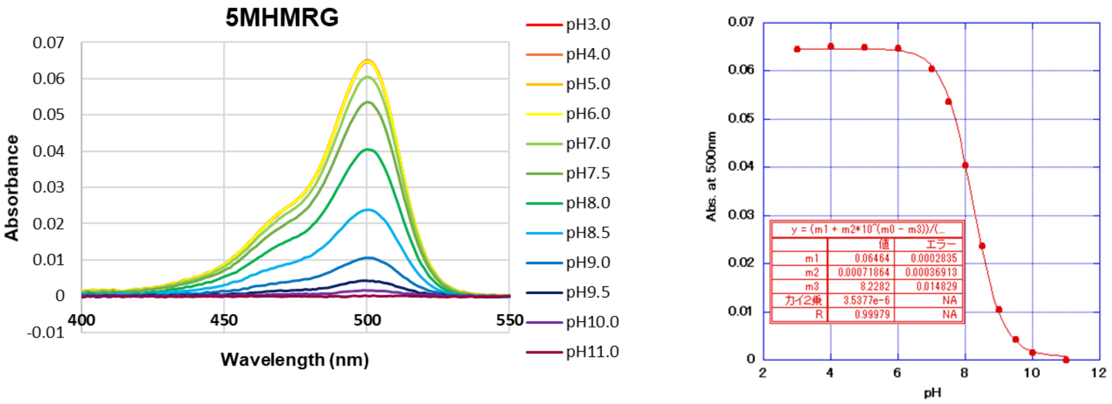

Supplementary Figure 19 Absorption spectra of 1 μM 5MHMRG, and correlation between absorbance at 505 nm and pH in 0.1 M NaPi buffer containing 0.1% DMSO as a cosolvent.

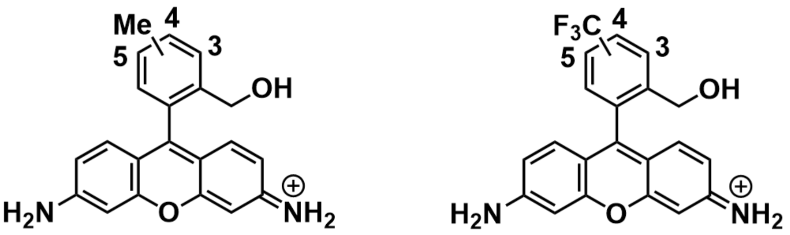

	3-Me	4-Me	5-Me
Open form	-1453.239658	-1453.244653	-1453.243302
Closed form	-1453.23629	-1453.237249	-1453.237625

	3-CF_3	4-CF_3	5-CF_3
Open form	-1750.984035	-1750.987576	-1750.988663
Closed form	-1750.982368	-1750.984056	-1750.982288

Supplementary Table 5 Calculated free energy values (hartree) of HMRG derivatives substituted at the benzene moiety.

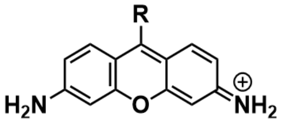

	R	Calculated pK_{cycl}
**HMRG**	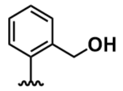	**7.9**
**THNaphtRG**	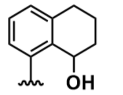	**8.5**
**HMCPRG**	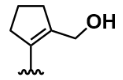	**12.1**
**HMCPDRG**	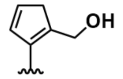	**13.8**

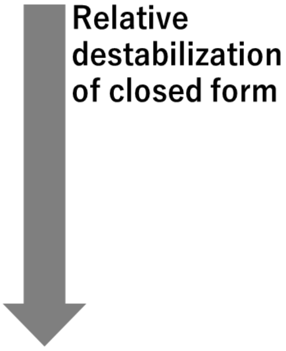

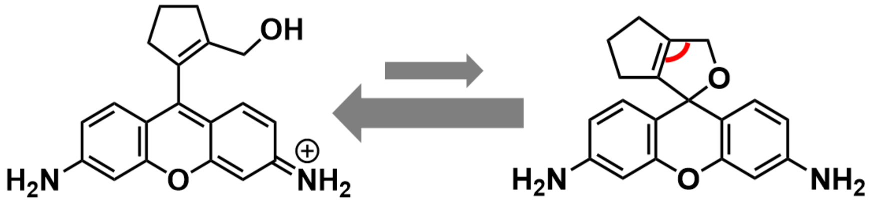

Supplementary Table 6 Ring-distortion effect on pK_{cycl}

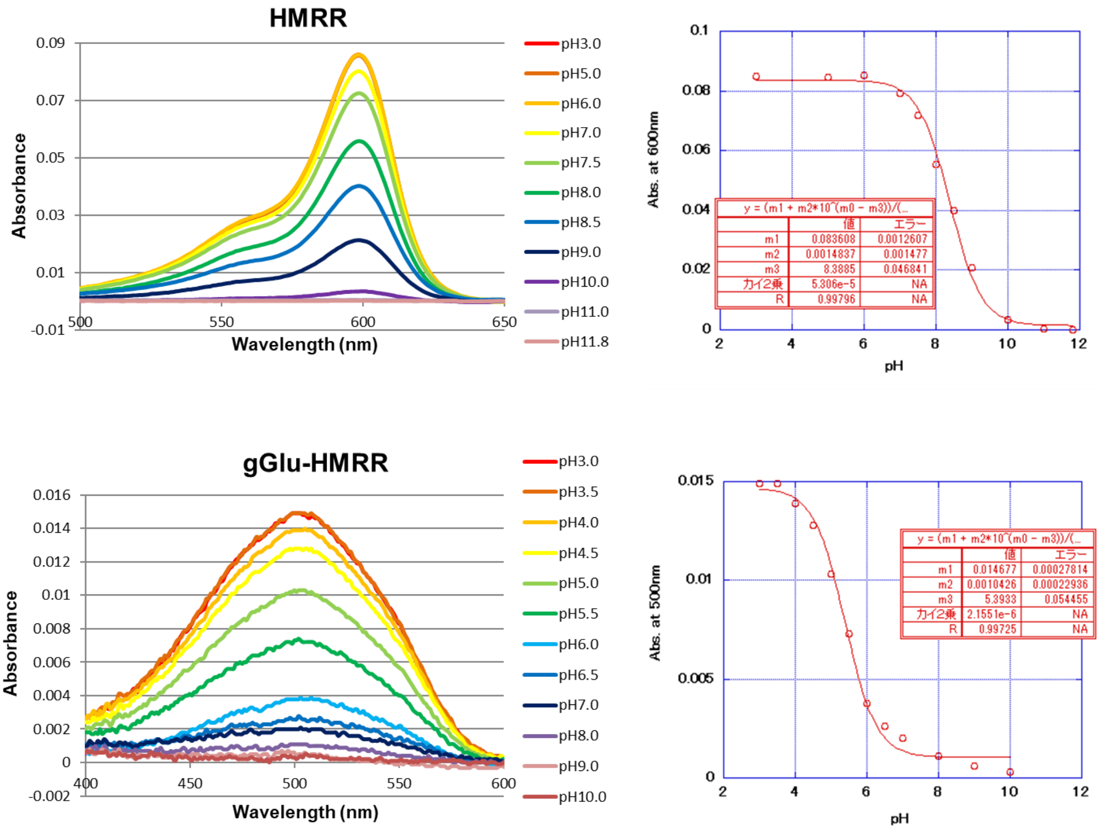

Supplementary Figure 20 Absorption spectra of 1 μM (gGlu-)HMRR, and correlation between absorbance at 600 (500) nm and pH in 0.1 M NaPi buffer containing 0.1% DMSO as a cosolvent.

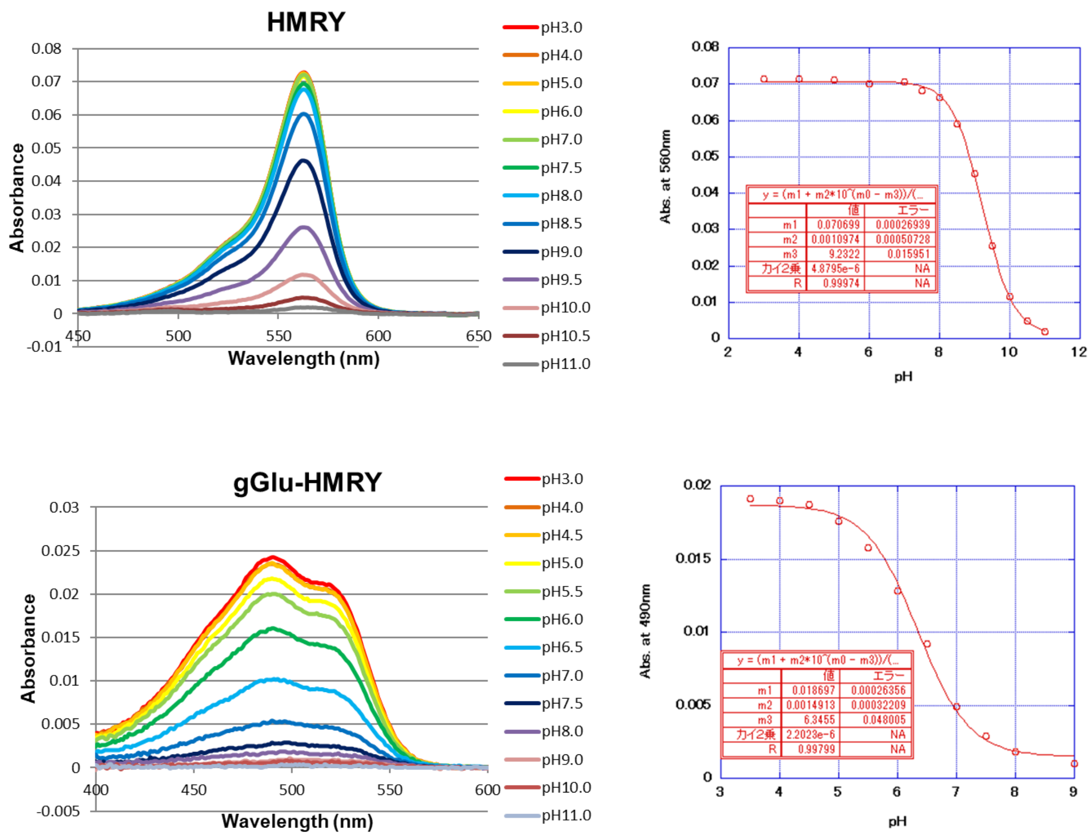

Supplementary Figure 21 Absorption spectra of 1 μM (gGlu-)HMRY, and correlation between absorbance at 560 (490) nm and pH in 0.1 M NaPi buffer containing 0.1% DMSO as a cosolvent.

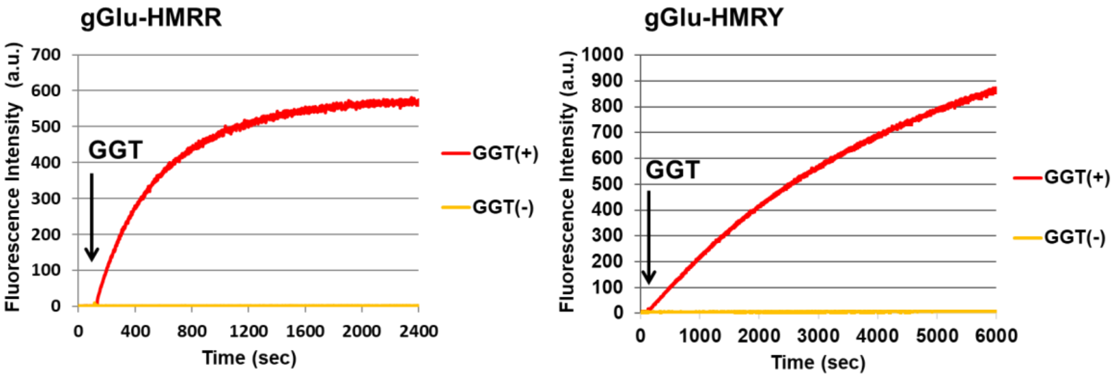

Supplementary Figure 22 Reaction of probes (3 μM) with GGT (1.1 U) in 10 mM NaPi buffer (pH 7.4) containing 0.03% DMSO as a cosolvent. GGT was added at 120 sec. Excitation, 593 nm (gGlu-HMRR), 550 nm (gGlu-HMRY) ; emission, 613 nm (gGlu-HMRR), 585 nm (gGlu-HMRY).

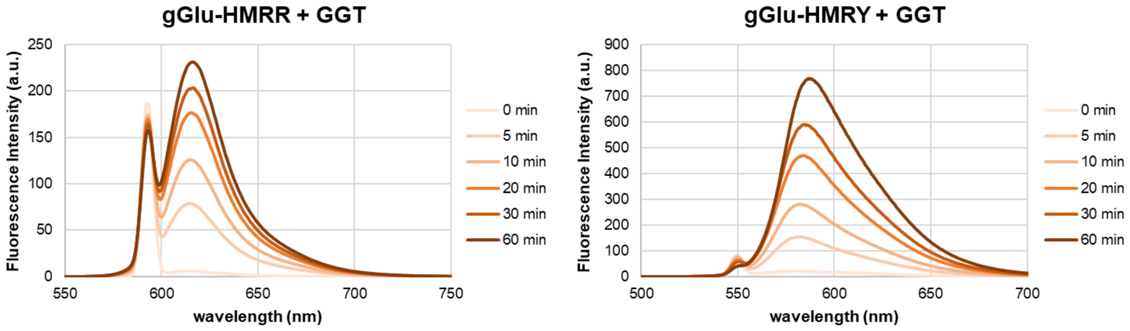

Supplementary Figure 23 Change of spectra in reaction of probes (3 μM) with GGT (1.1 U) in 10 mM NaPi buffer (pH 7.4) containing 0.03% DMSO as a cosolvent. Excitation, 593 nm (gGlu-HMRR), 550 nm (gGlu-HMRY).

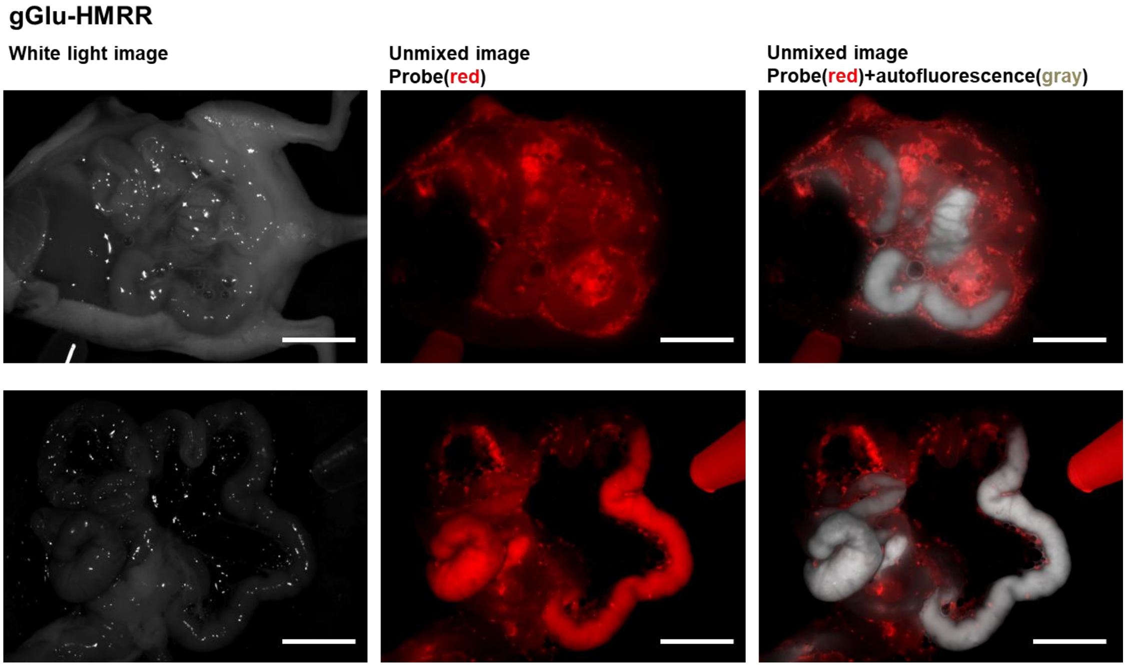

Supplementary Figure 24 Fluorescence spectral imaging of mouse models of peritoneal metastases at 5 min post-treatment with gGlu-HMRR (100 μM, 300 μL). In unmixed images, the probe fluorescence and autofluorescence were assigned as red and gray, respectively. Excitation, 575 to 605 nm; emission, 645 nm long-pass. Scale bar: 1 cm.

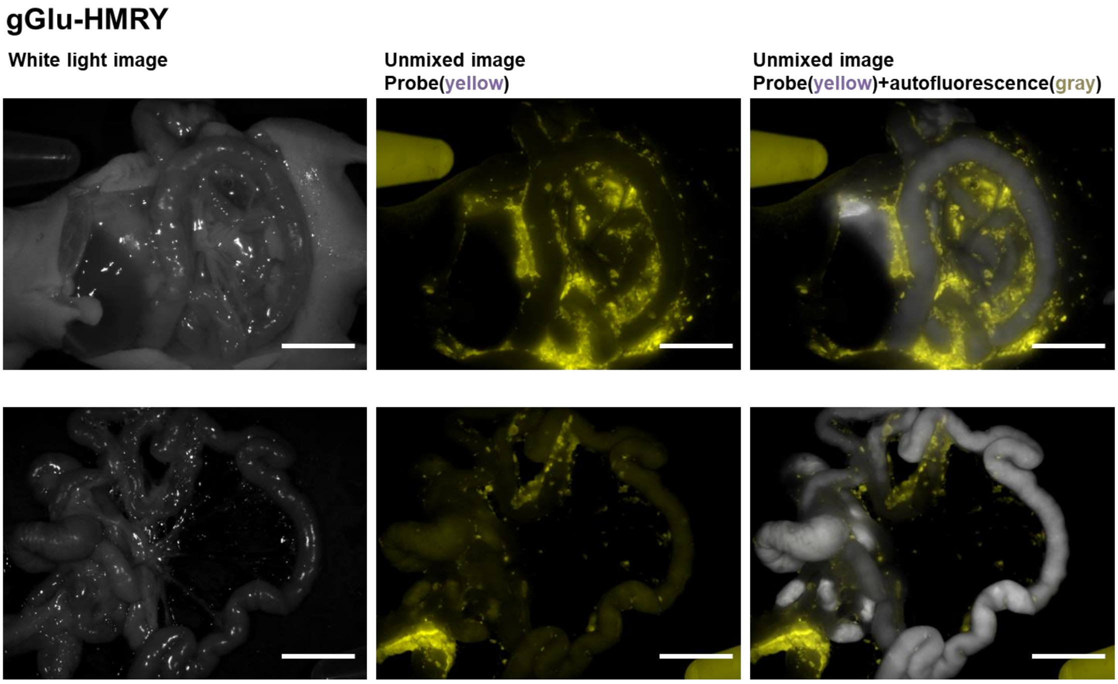

Supplementary Figure 25 Fluorescence spectral imaging of mouse models of peritoneal metastases at 5 min post-treatment with gGlu-HMRY (100 μM, 300 μL). In unmixed images, the probe fluorescence and autofluorescence were assigned as yellow and gray, respectively. Excitation, 503 to 555 nm; emission, 580 nm long-pass. Scale bar: 1 cm.

	After reaction with GGT		
	Emission maximum (nm)	φ_{fl}	S / N
gGlu-HMRR	616	0.26	> 500
gGlu-HMRY	587	0.58	~ 200
gGlu-HMJSiR *	662	0.20	~ 145
gGlu-HMJCR **	582	0.42	~ 30

Supplementary Table 7 Comparison of photochemical properties of spirocycle-based GGT probes (*cited from the literature[2], **cited from the literature[3]).

Supplementary References

1. Grimme, S., Antony, J., Ehrlich, S. & Krieg, H. A consistent and accurate ab initio parametrization of density functional dispersion correction (DFT-D) for the 94 elements H-Pu. *J. Chem. Phys.* **132**, (2010).
2. Iwatate, R. J. *et al.* Silicon Rhodamine-Based Near-Infrared Fluorescent Probe for γ-Glutamyltransferase. *Bioconjug. Chem.* **29**, 241–244 (2018).
3. Iwatate, R. J., Kamiya, M. & Urano, Y. Asymmetric Rhodamine-Based Fluorescent Probe for Multicolour in Vivo Imaging. *Chem. - A Eur. J.* **22**, 1696–1703 (2016).
