# Supplementary material for: Molecular design strategy of fluorogenic probes based on quantum chemical prediction of intramolecular spirocyclization
Source: Commun Chem. 2020 Jun 26;3:82. doi: 10.1038/s42004-020-0326-x (PMC9814528; doi:10.1038/s42004-020-0326-x)
Supplement: Supplementary file 2 — Description of Additional Supplementary Files [file 42004_2020_326_MOESM2_ESM.pdf]

Description of Additional Supplementary Files

File Name: Supplementary Data
Description: Cartesian coordinates of the optimized structures used in pKcycl calculation
